# Reproductive‐Triggered Sterol Competition Exacerbates Age‐Related Intestinal Barrier Damage in *Drosophila* Females

**DOI:** 10.1111/acel.70011

**Published:** 2025-02-07

**Authors:** Guixiang Yu, Kejin Chen, Mingyao Yang, Qi Wu

**Affiliations:** ^1^ Key Laboratory of Luzhou City for Aging Medicine, Department of Pharmacology, School of Pharmacy Southwest Medical University Luzhou China; ^2^ Institute of Animal Genetics and Breeding Sichuan Agricultural University Chengu China; ^3^ Central Nervous System Drug Key Laboratory of Sichuan Province Luzhou China

**Keywords:** *Drosophila*, intestinal barrier, reproduction, sterol

## Abstract

The trade‐off between reproduction and lifespan has been documented across a wide array of organisms, ranging from invertebrates to mammals. In malnourishing dietary conditions, inhibition of the reproductive processes generally extends the lifespan of females. However, the underlying mechanisms through which nutritional competition driven by reproduction accelerates aging remain poorly understood. Here, using female *Drosophila melanogaster* as a model, we show that among various dietary conditions lacking specific nutrients, only sterol deficiency significantly exacerbated both the incidence and severity of intestinal barrier deterioration during aging. Sterile mutation specifically ameliorated such damage in sterol‐deprived diets, but failed to alleviate age‐related intestinal barrier deterioration under other nutritional conditions. Additionally, we demonstrate that the lifespan extension and intestinal barrier amelioration, accompanied by a reproductive suppression effect, through the pharmacological inhibition of mTOR or Ras–Erk signaling using rapamycin or trametinib, were significantly modulated by cholesterol levels. Our study also identifies the morphological changes in excreta as a sensitive biomarker for early intestinal dysfunction. Collectively, these results suggest that the impairment of the intestinal barrier caused by reproductive‐induced sterol competition constitutes a significant factor limiting female lifespan in nutritionally unbalanced diets. This work elucidates a salient aspect of the complex interplay between reproductive resource allocation and somatic maintenance, thereby enhancing our understanding of how diet impacts the aging process.

## Graphical abstract

Dietary sterols are essential nutrients for maintaining intestinal barrier integrity. Sterol competition caused by female reproduction exacerbates intestinal barrier damage during aging, thereby shortening female lifespan.

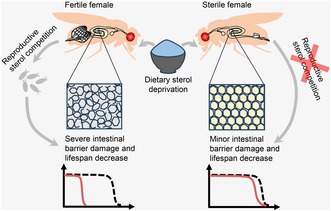

## 1 Introduction

Lifespan extension frequently tends to be accompanied by a reduction in female fecundity across a variety of organisms, a phenomenon particularly evident in longevity mediated by nutritional interventions. (Abe et al. 2024; Flatt et al. 2008; Hsin and Kenyon 1999; Long and Zhang 2023; Maklakov and Immler 2016; Min et al. 2012). In both invertebrates and vertebrates, lifespan is typically maximized under dietary conditions with a low protein‐to‐carbohydrate (P:C) ratio, whereas reproductive function is optimized in diets with a relatively higher P:C ratio. Therefore, this trade‐off is often interpreted as the result of differences in dietary nutrient composition required for optimizing reproduction and survival, rather than direct competition for resources between reproductive processes and somatic maintenance (Adler et al. 2013; Bock et al. 2019; Lee et al. 2008; Solon‐Biet et al. 2015). However, in the context of extreme nutritional imbalances—where the diet deviates from the nutrient composition that would optimally support either reproduction or longevity, a reduction in reproductive investment through genetic manipulation does substantially increase lifespan (Wu, Yu, Cheng, et al. 2020). This suggests that reproduction‐induced competition for essential nutrients is indeed a key factor limiting lifespan, at least in the case of malnutrition. Despite recognition of this interplay, the underlying mechanisms by which nutritional competition caused by reproduction reduces lifespan remain elusive and poorly characterized.

*Drosophila melanogaster* serves as a classic model organism in the fields of nutritional physiology and aging research. Our previous studies have shown that reproduction‐induced competition for limited nutrients significantly decreased female lifespan in the context of an unbalanced diet (Wu, Yu, Cheng, et al. 2020). Furthermore, this reproductive‐linked nutritional competition extends beyond essential macronutrients such as carbohydrates and proteins. Recent evidence indicate that the competition for critical non‐energy nutrients, including cholesterol and vitamins, caused by reproductive activities, also significantly contributes to the decrease of female lifespan (Wu, Yu, Cheng, et al. 2020; Yu et al. 2023; Zanco et al. 2021). One potential explanation is that this competition leads to reduced resource allocation to somatic maintenance, thereby compromising the integrity of various tissues and organs (Maklakov and Immler 2016). However, the specific tissue or organ pathologies linked to reproduction induced nutritional competition, as well as the relative contributions of different nutrients to these processes, has yet to be fully elucidated.

The interplay between the gastrointestinal and reproductive systems plays a crucial role in maintaining overall organism health and enhancing reproductive efficiency. A quintessential and primary mode of interaction between the gut and ovaries manifests through the adjustment of reproductive output contingent upon nutritional status (Drummond‐Barbosa and Spradling 2001; Terashima and Bownes 2004). Besides that, signaling from the gut to ovaries is essential for germline stem cell proliferation and subsequent egg production postmating (Ameku et al. 2018). The morphological and physiological changes in the female gut after mating also contribute to the maintenance of normal reproductive function (Reiff et al. 2015; White et al. 2021). Moreover, steroid hormone ecdysone from the ovaries to the intestine also promotes intestinal growth and improves the reproductive output of females, but at the same time increases the pathology of the intestine with age (Ahmed et al. 2020). Elucidating the intricacies of the gut‐ovary axis in *Drosophila melanogaster* not only sheds light on the evolutionarily conserved mechanisms underpinning the interactions between digestive and reproductive systems but also may unveil novel insights into the overarching regulatory networks orchestrating physiological processes. In this study, we demonstrate that competition for sterol triggered by reproductive activity exacerbates age‐associated deterioration of the intestinal barrier in female *Drosophila*.

## 2 Results

### 2.1 Female Reproduction Exacerbates the Intestinal Barrier Dysfunction Caused by Cholesterol Deficiency

**FIGURE 1:**
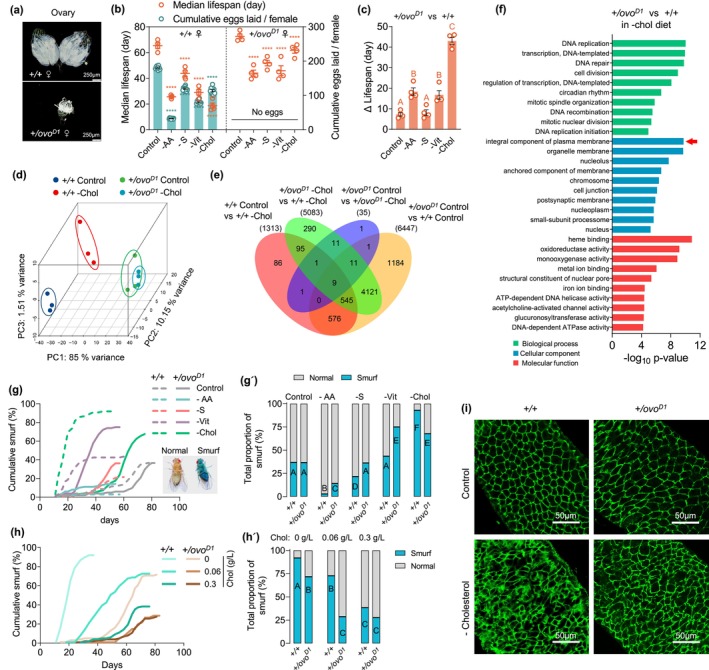
Reproductive‐triggered cholesterol competition exacerbates age‐related intestinal barrier damage in females. (a) Ovary of *Dahomey* wild‐type females (*+/+* ♀) and *Dah; ovo* ^*D*1^ infertile mutant females (*+/ovo* ^*D*1^ ♀). (b) Effects of omitting dietary amino acids (AA), sucrose (S), B‐group vitamins (Vit) or cholesterol (Chol) on fecundity and median lifespan of *+/+* ♀ and *+/ovo* ^*D*1^ ♀. (*n* = 10 biological replicates for egg laying in each treatment. Egg numbers in 24‐h periods were counted every 2–3 days from day 1 to day 17, and are reported as cumulative eggs laid per female. *n* = 4 replicates for median lifespan in each treatment, each replicate contained 100 flies per treatment. Error bars represent mean ± s.e.m. ****p < 0.0001, versus control group. Lifespan differences were assessed using the Cox regression model. Egg‐laying differences were assessed by one‐way ANOVA followed by Tukey's multiple comparison. See statistical analysis of fecundity data in Table S1) (c) Δ lifespan between *+/ovo* ^*D*1^ ♀ and *+/+* ♀ (Median lifespan of *+/ovo* ^*D*1^ ♀ minus Median lifespan of *+/+* ♀) in control and specific nutrients omitted diets. The survival advantage of *+/ovo* ^*D*1^ ♀ relative to *+/+* ♀ is most prominent in the cholesterol‐deficient diet. (Unique letters above the data indicate significant differences between conditions, *p* < 0.05. paired *t*‐test. Bars represent mean ± s.e.m.) (d) Three‐dimensional PCA based on the differentially expressed genes of *+/+* ♀ and *+/ovo* ^*D1*^ ♀ that fed with control or cholesterol‐omitted diet. (e) Venn diagram of differentially expressed genes. (f) Top 30 GO terms of the DEGs between *+/+* ♀ and *+/ovo* ^*D1*^ ♀ in cholesterol dropout diet. (g–g′) *+/ovo* ^*D1*^ mutation significantly improved the intestinal barrier integrity of females in cholesterol dropout diet but not in other nutritional conditions. Cumulative percentage (g) and total proportion (g′) of “smurf” flies in *+/+* ♀ and *+/ovo* ^*D1*^ ♀ fed with control or specific nutrients omitted diets. (h–h′) Effects of dietary cholesterol concentration on cumulative percentage (h) and total proportion (h′) of “smurf” flies in *+/+* ♀ and *+/ovo* ^*D1*^ ♀. (For “smurf” assay in panel g–h′, *n* = 96 ~ 103 flies in each treatment. Age‐related cumulation of ‘smurf’ were assessed using the Cox regression model. The interactions of diets and genotype were significant in all nutrient manipulations (*p* < 0.05, Cox regression). Total proportion of “smurf” were assessed by Fisher's exact test. Unique letters above the data indicate significant differences between conditions, *p* < 0.05. See statistical analysis of “smurf” data in Tables S3 and S8) (i) *+/ovo* ^*D*1^ ♀ have better intestinal cell membrane integrity than *+/+* ♀ after fed with cholesterol dropout diet for 10 days.

Amino acids, sugars, sterols, and B‐group vitamins are the most crucial dietary components that substantially dictate the lifespan of female flies (Piper et al. 2014; Wu, Yu, Cheng, et al. 2020). Consistent with our previous studies (Wu, Yu, Cheng, et al. 2020), *ovo* ^*D*1^ sterile mutant females in which oogenesis is blocked at stage 4 (Granadino et al. 1992), lived longer than wild‐type females under various conditions of nutrient deprivation (Figure 1a,b and Figure S1a,b, Table S1). Significantly, the survival advantage of *ovo* ^*D*1^ mutants was markedly greater under conditions of cholesterol (represents sterols in FLYaa chemically defined medium) deficiency than with other nutritional limitations (Figure 1c). It suggests that competition for cholesterol, driven by reproductive demands, is the predominant factor decreasing female lifespan under nutritional deficiencies.

To investigate how reproductive‐induced cholesterol competition contributes to shortened female lifespan, we performed whole‐fly transcriptomic analyses of wild‐type females and *ovo* ^*D*1^ females that were fed with either a nutritionally complete diet or a cholesterol dropout diet. Interestingly, significant inherent differences in gene expression existed between wild‐type and *ovo* ^*D*1^ females on both diets, revealing distinct response patterns to cholesterol deprivation. Consistent with the effect of dietary cholesterol omission on lifespan, principal‐component analysis (PCA) of differentially expressed genes (DEGs) showed that the effect of cholesterol absence on wild‐type females was much greater than that on *ovo* ^*D*1^ mutant females (Figure 1d). Specifically, cholesterol deprivation significantly altered the expression of 1313 genes in wild‐type females, contrasting sharply with the modulation of only 35 genes in *ovo* ^*D*1^ mutants (Figure 1e). Gene Ontology (GO) enrichment analysis showed that the DEGs of wild‐type and *ovo* ^*D1*^ females on a cholesterol‐deficient diet were involved in a very wide range of metabolic processes. Among them, one of the most significant transcriptional changes is associated with the components of the plasma membrane (Figure 1f), which is consistent with the role of cholesterol as an essential component in cellular membrane composition (Krause and Regen 2014).

The intestine of *Drosophila*, made up of epithelial monolayers of enterocytes, is highly susceptible to disruptions in cell membrane integrity, thus influencing both structure and function significantly (Colombani and Andersen 2020). Therefore, to investigate whether the significant decrease in the lifespan of wild‐type females compared to *ovo* ^*D1*^ females on a cholesterol deficiency diet, is attributable to compromised intestinal barrier integrity, we tested the effects of omitting cholesterol or several other nutrients from the nutritionally complete medium on the incidence of “smurf” flies. “Smurf” refers to the condition in flies where a non‐absorbed blue dye (FD&C blue no. 1), ingested with food, diffuses throughout the body from the intestine, indicating compromised intestinal barrier integrity. Interestingly, although omitting any of these nutrients greatly decreased lifespan (Figure S1b, Table S2), and the cumulative number of “smurf” flies increased with age under all nutritional conditions, the extent of the impact on the intestinal barrier varied by nutrient (Figure 1g,g′, Table S3). For wild‐type females, neither sugar nor amino acids deprivation in the diet increased the total proportion of ‘smurf’ compared to the control group. Vitamin deficiency significantly accelerated the onset of intestinal barrier impairment without increasing the cumulative proportion of “smurf” flies. Distinct from the effects observed with other nutrients, the absence of dietary cholesterol not only significantly advanced the time of intestinal barrier damage but also greatly increased the proportion of accumulated “smurf” flies over the life history (Figure 1g,g′, Table S3). Compromised lifespan and intestinal barrier integrity due to cholesterol deficiency was also consistent in two other strains, *w* ^1118^ and *Canton S* (Figure S1d,g, Tables S4–S7). Moreover, relative to cholesterol‐rich diet condition (0.3 g/L cholesterol), simply reducing dietary cholesterol concentration, rather than removing cholesterol completely, also increased the proportion of “smurf” and decreased lifespan (Figure 1h,h′ and Figure S1c, Tables S8 and S9), suggesting a causal link between cholesterol deficiency and exacerbated intestinal barrier degradation.

Surprisingly, although the *ovo* ^*D*1^ mutation reduced the nutritional competition associated with reproduction, it led to an increased occurrence of the “smurf” phenotype throughout the lifespan of female flies on diets lacking amino acids, sucrose, or vitamins. In contrast, only in a cholesterol‐deficient diet did *ovo* ^*D*1^ mutations both significantly delayed the onset of the intestinal barrier damage and reduced the cumulative incidence of “smurf” flies (Figure 1g,g′, Table S3). In addition, dietary cholesterol deprivation in adult stage had no effect on male lifespan or age‐related intestinal barrier damage. And virgin females that laid fewer eggs than mated females also lived longer, and had a later onset time of the explosive increase of “smurf” flies on a diet without cholesterol (Figure S1h,k, Tables S10–S12). Moreover, the concentrations of cholesterol required to sustain both the full lifespan and intestinal integrity in *ovo* ^*D1*^ females are comparatively lower than those required in wild‐type females (Figure 1h,h′ and Figure S1c, Tables S8 and S9). Corroborating these observations, *ovo* ^*D*1^ mutations also mitigated intestinal pathological damage caused by cholesterol deprivation, which is mainly characterized by disordered cell arrangement and broken cell membranes (Regan et al. 2016; Resnik‐Docampo et al. 2017). (Figure 1i). Collectively, all these data suggest that reducing the cholesterol competition caused by reproduction significantly improved the intestinal barrier function in females.

### 2.2 Sterols, Not Fatty Acids or Steroid Hormones, Are Necessary for the Integrity of the Intestinal Barrier in Female *Drosophila*

**FIGURE 2:**
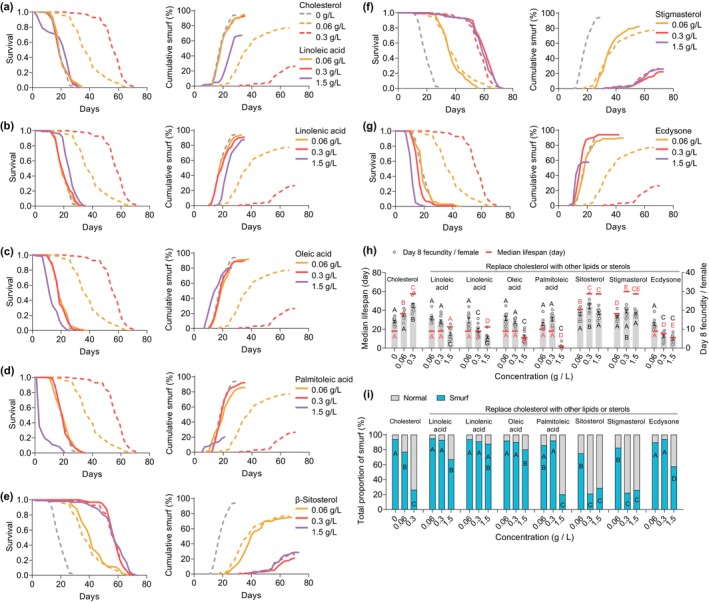
Phytosterols, but not fatty acids or steroid hormones, restored longevity, reproduction, and intestinal barrier integrity in females on a cholesterol‐deficient diet. (a–g) Effects of linoleic acid (a), linolenic acid (b), oleic acid (c), palmitoleic acid (d), sitosterol (e), stigmasterol (f), or ecdysone (g) on female lifespan and age‐related cumulative “smurf” ratio in a cholesterol‐deficient diet. (h–i) Median lifespan, day 8 fecundity (h), and total proportions of “smurf” (i) of females fed different doses of cholesterol, linoleic acid, linolenic acid, oleic acid, palmitoleic acid, sitosterol, stigasterol, or ecdysone. (*n* = 100 flies per treatment for lifespan and “smurf” assay, *n* = 10 biological replicates for egg laying in all trials, error bars represent mean ± s.e.m. For panel h and i, unique letters above or below the data indicate significant differences between cholesterol and other treatment groups, *p* < 0.05. Lifespan differences were assessed using the Cox regression model. Egg‐laying differences were assessed by one‐way ANOVA followed by Tukey's multiple comparisons test. For panel a–g, age‐related cumulation of “smurf” were assessed using the Cox regression model. For panel i, total proportion of “smurf” flies were assessed by Fisher's exact test. See statistical analysis of lifespan, “smurf” and fecundity data in Tables S13–S16).

In addition to sterols, previous studies have shown the essential role of fatty acids in maintaining cell membrane integrity (de Carvalho and Caramujo 2018; Mukerjee et al. 2021). Given the absence of fatty acids in FLYaa medium, we further investigated whether fatty acids could effectively substitute for cholesterol in preserving the integrity of the female intestinal barrier. Contrary to the beneficial effects of cholesterol on longevity and intestinal barrier integrity, substituting cholesterol in the medium with equivalent doses of either polyunsaturated fatty acids (linoleic acid or linolenic acid) or monounsaturated fatty acids (palmitoleic acid or oleic acid) failed to mitigate the lifespan reduction and intestinal barrier damage (referred to as “smurf”) associated with cholesterol deficiency. Although higher concentrations of polyunsaturated fatty acids slightly extended female lifespan and reduced the “smurf” phenotype on a cholesterol‐free diet, they significantly decreased female fecundity (Figure 2a–c,h,i, Tables S13–S16). Similarly, elevated levels of monounsaturated fatty acids decreased the overall “smurf” proportion but concurrently led to reduced lifespan and fecundity (Figure 2a,d,h,i, Tables S13–S16). In contrast, the substitution of dietary cholesterol with the phytosterols stigmasterol and sitosterol completely restored female lifespan, fecundity, and intestinal barrier integrity to levels comparable to cholesterol‐rich diets (Figure 2e,f,h,i, Tables S13–S16). Given that *Drosophila* can convert sterol precursors into the steroid hormone ecdysone, which is important for the development of numerous organs (Ahmed et al. 2020; Yamanaka et al. 2013), we examined whether the intestinal barrier damage induced by sterol deficiency is linked to impediments in ecdysone synthesis. We found that supplementation with ecdysone in a cholesterol‐deficient medium fails to recover female longevity or fecundity. Furthermore, similar to the effects of monounsaturated fatty acids, high doses of ecdysone reduced “smurf” accumulation but resulted in significant lifespan reduction and decreased fecundity (Figure 2g–i, Tables S13–S16). Therefore, our study indicates that sterols, rather than fatty acids or steroid hormones, are indispensable for the maintenance of intestinal barrier integrity in female flies.

### 2.3 Diarrhea Is an Early Phenotype of Intestinal Dysfunction Caused by Sterol Deficiency

**FIGURE 3:**
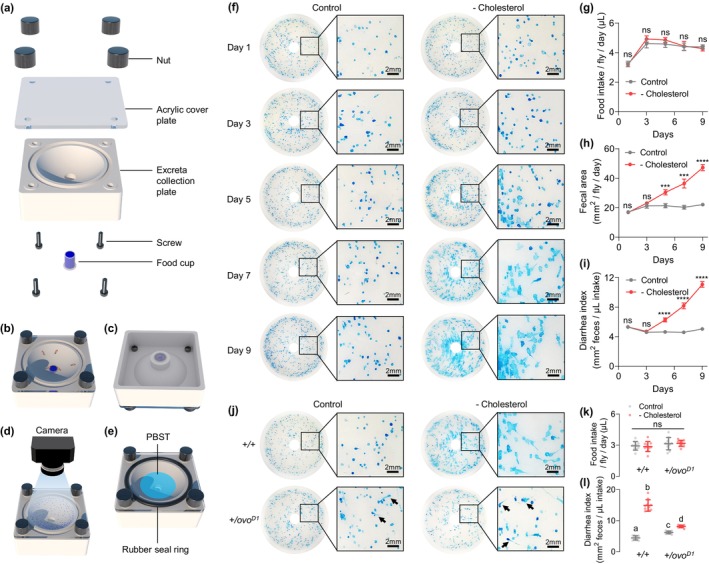
Reproductive‐triggered sterol competition causes diarrhea in females. (a–e) Assembly and usage procedures of the excreta‐quantification (EX‐Q) chamber for simultaneous measurement of excreta area and food intake in *Drosophila*. (a) Assembly diagram of the EX‐Q chamber. (b, c) The front (b) and back (c) of the assembled EX‐Q chamber. Transfer 5 flies to each chamber and feed for 24 h. (d) After 24 h, remove the food cup and transfer flies out of the EX‐Q chamber, photograph the excreta collection plate. (e) Insert a rubber plug into the hole at the bottom of the excreta collection plate, place a rubber seal ring in the annular groove, and then add 2 mL PBST to the plate to dissolve the dye in the excreta. Food intake is calculated by measuring the absorbance of the solution. (f) Representative fecal photos of wild *Dahomey* females from day 1 to day 9 under nutritionally complete diet or cholesterol dropout diet feeding conditions. (g) Omitting dietary cholesterol did not affect the food intake of wild *Dahomey* females. (h–i) Cholesterol omission caused a distinct diarrheal phenotype from day 5 onwards, including increased fecal area (h) and diarrhea index which defined as mm^2^ feces/μL food intake (i). (j) Representative fecal photos on day 10 of *Dahomey* wild‐type females (*+/+*) and *Dah; ovo* ^*D*1^ mutant females (*+/ovo* ^*D*1^) under nutritionally complete diet or cholesterol dropout diet feeding conditions. The black arrow points to irregularly shaped and trailing feces of *ovo* ^*D*1^ females. (k) Cholesterol omission had no significant effect on food intake of wild type and *ovo* ^*D1*^ females. (l) *ovo* ^*D*1^ mutation slightly increased the diarrhea index of females under nutritionally complete feeding condition, but greatly reduced the diarrhea index of females that fed with cholesterol dropout diet. (For all excreta quantification and food intake measurement, *n* = 10 replicates in each treatment, each replicate contained 5 flies. Error bars represent mean ± s.e.m. For panel h–i, ***p < 0.001, ****p < 0.0001. Assessed by unpaired t test. For panel l, unique letters above the data indicate significant differences between conditions, *p* < 0.05. Assessed by two‐way ANOVA followed by Sidak's multiple comparisons test.)

Previous studies indicated that the morphological characteristics of *Drosophila* excreta were closely related to the physiological state of the intestine (Cognigni et al. 2011; Wayland et al. 2014). Therefore, we further investigated the changes in fecal morphology and area of females under cholesterol‐deficient diet conditions and whether it can be used as an early pathologic phenotype reflecting sterol deficiency. Considering the direct relationship between excreta volume and food consumption, in order to measure food intake and fecal area of *Drosophila* simultaneously, we modified our previously established excreta‐quantification (EX‐Q) assay by introducing a novel apparatus (Figure 3a). Briefly, to maximize fecal collection efficiency without disrupting normal feeding behavior, flies were housed for 24 h in a chambered fecal collection plate in which a small food cup containing dye‐labeled food was mounted at the bottom. Subsequently, the flies were removed, and the feces collected on the plate were imaged to evaluate their area and morphology. The dye from the collected feces was then solubilized in PBST to measure absorbance and derive a quantification of food intake, thereby enabling the calculation of fecal area per unit of consumed food (Figure 3a–e, Figure S2a–d).

Our previous studies have indicated that foods with a minimal surface area (~6 mm diameter) utilized in the EX‐Q assay do not influence the food intake of *Drosophila* (Wu, Yu, Park, et al. 2020). To ascertain whether extended rearing periods within the EX‐Q box impact the normal physiology of the flies, we conducted a comparative analysis of body weight, triglyceride levels, and cholesterol concentrations in flies reared for 10 days in both EX‐Q boxes and standard *Drosophila* vials. Our analysis revealed no significant differences in these physiological parameters between the two feeding conditions, suggesting that prolonged exposure to EX‐Q feeding does not detrimentally affect *Drosophila* physiology (Figure S2e).

Using the EX‐Q assay, we demonstrated that sterol deprivation did not affect food consumption in females (Figure 3g,k). However, significant changes in the fecal morphology of wild‐type females were detected as early as day 5 following dietary cholesterol deprivation. Characteristics such as increased fecal area, lighter coloration indicating higher water content, more irregular shape, and significant trailing are typical of the feces from wild‐type females under conditions of cholesterol deficiency, aligning with a “diarrhea” phenotype (Liu and Chassagne 2023) (Figure 3f,h,i). Interestingly, on the full‐nutrient diet, both the fecal area and morphological irregularities in *ovo* ^*D*1^ females were slightly greater than those observed in wild‐type females, despite no differences in food intake (Figure 3j–l). This is consistent with the slower transit and longer retention of food in the intestines of wild‐type females, resulting in more concentrated feces (Cognigni et al. 2011). Nevertheless, the “diarrhea” symptoms were considerably milder in *ovo* ^*D*1^ females than in wild‐type females when subjected to a cholesterol‐deficient diet (Figure 3j–l). These suggest that cholesterol competition triggered by reproductive processes does exacerbate intestinal dysfunction in females, in which “diarrhea” can be identified as an early phenotype.

### 2.4 Pharmacological Inhibition of mTOR or Ras–Erk Signaling Pathways Mitigates Intestinal Barrier Damage Caused by Sterol Deficiency

**FIGURE 4:**
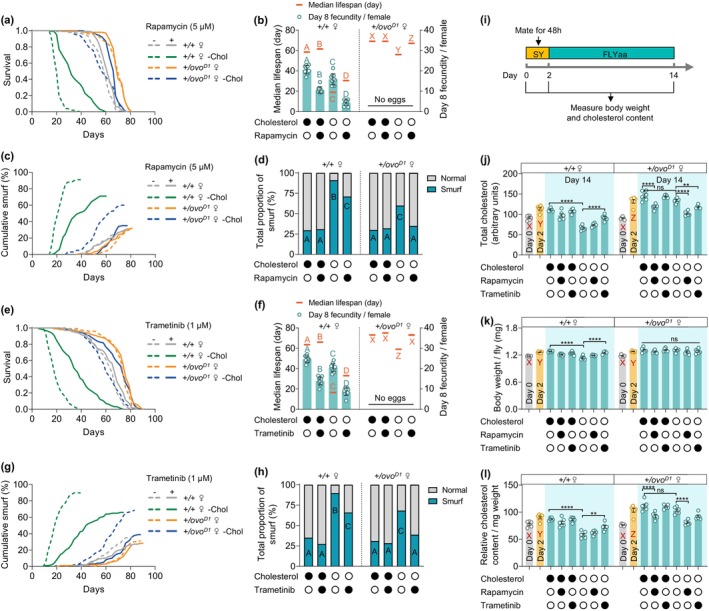
Cholesterol dose‐dependence of rapamycin and trametinib in improving intestinal barrier function in females. (a, b, e, and f) Rapamycin (a, b) and trametinib (e, f) significantly decreased fecundity of *Dahomey* wild‐type females (*+/+* ♀) (b, f) and greatly increased lifespan (a, e) of both *+/+* ♀ and *Dah; ovo* ^*D*1^ mutant females (*+/ovo* ^*D*1^ ♀) in cholesterol dropout diet. (c, d, g, and h) Rapamycin (c, d) and trametinib (g, h) significantly improved intestinal barrier function of both *+/+* ♀ and *+/ovo* ^*D*1^ ♀ in a cholesterol‐deficient diet. (i) Experimental timeline for body weight and cholesterol content measurements of experimental flies. (j–l) Effects of rapamycin and trametinib on total cholesterol levels (j), body weight (k) and cholesterol abundance (l) in wild‐type and *ovo* ^*D*1^ infertile females fed a cholesterol‐rich or cholesterol‐free diet. (For panels b, d, f, h, j–l, solid black dots below the x‐axis indicate the presence of cholesterol, rapamycin or trametinib in the medium, while open circles indicate their absence. *n* = 100 flies per treatment for lifespan and “smurf” assay, *n* = 10 biological replicates for egg laying, *n* = 6 biological replicates for body weight and cholesterol content measurements in all trials, error bars represent mean ± s.e.m. For panel b, d, f, h and j–l, unique letters above or below the data indicate significant differences between conditions, *p* < 0.05. Lifespan differences were assessed using Cox regression model. Egg‐laying differences were assessed by two‐way ANOVA followed by Tukey's multiple comparisons test. For panel c and g, age‐related cumulation of “smurf” were assessed using the Cox regression model. For panel d and h, total proportion of “smurf” flies were assessed by Fisher's exact test. For panel j–l, dody weight, cholesterol level and cholesterol abundance were assessed by Multivariate ANOVA followed by Tukey's multiple comparisons. **p < 0.01, ****p < 0.0001. See statistical analysis of lifespan, “smurf” and fecundity data in Tables S17–S19, S22–S24, and S28–S30).

The inhibition of either the mTOR or Ras–Erk signaling pathway extends the lifespan of fruit flies and improves age‐related intestinal barrier damage, however, it is also accompanied by a significant decrease in female fecundity (Bjedov et al. 2010; Fan et al. 2015; Slack et al. 2015; Ureña et al. 2024). Nevertheless, it remains unclear whether their ameliorative effects on intestinal barrier integrity are indirectly mediated through the suppression of reproductive functions and the subsequent redistribution of sterol between reproduction and somatic maintenance. To elucidate this, we investigated the effects of classical inhibitors of these two pathways, rapamycin and trametinib, on the lifespan and intestinal barrier integrity of wild‐type and *ovo* ^*D1*^ mutant females under varying dietary cholesterol conditions. Consistent with a previous study (Zanco et al. 2021), the effect of rapamycin on the lifespan of wild‐type females showed a significant cholesterol‐dependent effect, specifically, rapamycin increased lifespan in cholesterol‐deficient diets by a much greater magnitude than that in cholesterol‐rich dietary conditions (60.5% vs. 5.1%), despite a significant reduction in fecundity in both conditions. Furthermore, although rapamycin failed to increase the lifespan of *ovo* ^*D*1^ females in a nutritionally complete medium, it significantly increased their lifespan in a cholesterol dropout diet (Figure 4a,b, Tables S17 and S18). Additionally, rapamycin also significantly improved intestinal barrier function in both wild‐type and *ovo* ^*D*1^ mutants on a cholesterol‐deficient diet, but failed to ameliorate age‐related intestinal barrier damage in both genotypes under cholesterol‐abundant conditions (Figure 4c,d and Figure S4a, Table S19).

Previous studies have demonstrated that trametinib extends the lifespan of female flies when administered in a yeast‐sucrose (YS) medium (Castillo‐Quan et al. 2019; Slack et al. 2015; Ureña et al. 2024), however, its effects in chemically defined medium remain unclear. The food intake and drug absorption efficiency of flies were very different between YS and chemically defined medium (Piper et al. 2014; Wu, Yu, Park, et al. 2020). Therefore, drawing on the differential effective concentrations of rapamycin required for lifespan extension in YS medium (50–200 μM) compared to that in chemically defined medium (5–10 μM) (Bjedov et al. 2010; Wu et al. 2017), alongside the established optimal concentration of trametinib (15.6 μM) in YS medium (Castillo‐Quan et al. 2019; Slack et al. 2015; Ureña et al. 2024), we investigated the possible life‐extension concentration of trametinib in FLYaa chemically defined medium. Our results indicate that trametinib, in doses ranging from 0.5 μM to 2 μM, dose‐dependently reduced the fecundity of wild‐type female flies and concurrently extended their lifespan in FLYaa diets. Furthermore, 0.5 μM and 1 μM trametinib maximized lifespan under cholesterol‐rich and cholesterol‐deficient diets, respectively (Figure S3a,b, Tables S20 and S21). In subsequent experiments, 1 μM trametinib was utilized to assess both lifespan and intestinal barrier function, as it significantly reduced Erk phosphorylation under both cholesterol‐rich and cholesterol‐deficient diets (Figure S3c). Echoing the effects observed with rapamycin, trametinib also increased the lifespan of both wild‐type and *ovo* ^*D*1^ mutant females in a cholesterol‐dependent manner, whereas ameliorating intestinal barrier damage induced by cholesterol deficiency (Figure 4e–h and Figures S3d,g and S4a, Tables S22–S27). Most surprisingly, trametinib restored both the lifespan and intestinal barrier integrity of *ovo* ^*D*1^ females on the cholesterol‐deficient diet to levels comparable to those seen under nutritionally complete conditions (Figure 4e–h, Tables S22–S24).

Consistent with the ameliorated intestinal pathology (Figure S4a), rapamycin and trametinib also significantly alleviated the “diarrhea” phenotype caused by cholesterol deprivation in both wild‐type and *ovo* ^*D*1^ females (Figure S4b–e).

To elucidate whether rapamycin and trametinib‐induced amelioration in intestinal barrier integrity under cholesterol‐deprived dietary conditions are associated with alterations in in vivo cholesterol levels, we further investigated the effects of these two drugs on body weight and total cholesterol concentrations in both wild‐type and *ovo* ^*D*1^ females. Throughout our study, all experimental flies were reared on a yeast‐sucrose medium during development and for the initial 2 days post‐eclosion. Therefore, to determine whether cholesterol acquired during development and early adulthood contribute to the disparities in intestinal barrier damage observed under cholesterol‐deprived conditions between wild‐type and *ovo* ^*D*1^ mutants, we also measured both body weight and total cholesterol in females immediately post‐eclosion and after a 48‐h yeast‐sucrose medium exposure (Figure 4i). Our data indicate that prior to post‐eclosion feeding, total cholesterol levels do not significantly differ between wild‐type and *ovo* ^*D*1^ females. Following 48 h on the yeast medium, both body weight and total cholesterol increased markedly in both genotypes. Although body weight did not differ significantly between wild‐type and *ovo* ^*D*1^ females at this stage, cholesterol levels were slightly higher in *ovo* ^*D*1^ females (Figure 4j,k), possibly due to the avoidance of cholesterol consumption associated with egg‐laying, resulting in increased bodily cholesterol storage.

Notably, a cholesterol‐deprived diet administered for 12 days significantly reduced body weight, total cholesterol, and cholesterol abundance (cholesterol content per unit body mass) in wild‐type females but exerted no significant effect on *ovo* ^*D*1^ counterparts (Figure 4j–l, Tables S28–S30). Given that dietary cholesterol deprivation during developmental stages has been shown to mildly down‐regulate the insulin signaling level of Drosophila larvae, thereby delaying their developmental progression (Texada et al. 2022), we further investigated whether the reduction in body weight observed in wild‐type adult females due to dietary cholesterol deprivation is also related to the downregulation of insulin signaling. Unlike the effects caused by cholesterol deficiency during larval stages (Texada et al. 2022), dietary cholesterol deprivation in adults did not significantly alter the phosphorylation levels of Akt or the total ATP levels in vivo (Figure S3h,i). This suggests that the reduction in body weight resulting from adult‐stage cholesterol deprivation is a reproductive‐dependent nutritional metabolic disorder rather than one dependent on the insulin signaling pathway.

The impact of rapamycin and trametinib on female cholesterol levels demonstrated a strong dependency on reproductive status. Under both cholesterol‐sufficient and cholesterol‐deficient dietary settings, rapamycin exerted no significant influence on the body weight or total cholesterol in wild‐type females, yet it significantly decreased cholesterol levels in *ovo* ^*D*1^ females. In contrast, trametinib did not significantly alter cholesterol content in either genotype under nutritional complete dietary conditions but exhibited different effects under cholesterol deprivation, significantly elevated both body weight and cholesterol levels in wild‐type females whereas slightly reducing cholesterol levels in *ovo* ^*D*1^ females. Nevertheless, as dietary cholesterol deprivation alone did not lower cholesterol levels in *ovo* ^*D*1^ females, despite rapamycin and trametinib reducing cholesterol levels under cholesterol‐deprived conditions, *ovo* ^*D1*^ females maintained significantly higher total cholesterol levels and cholesterol abundance than wild‐type females within the same dietary framework (Figure 4j–l, Tables S28–S30).

All these data suggest that the effect of rapamycin and trametinib on intestinal barrier function appears to be confined to scenarios involving a cholesterol‐restricted diet. Notably, although the elimination of nutrient competition caused by reproduction did not block the improvement effect of both rapamycin and trametinib on intestinal barrier integrity under cholesterol‐deficient dietary conditions, the two drugs exhibited different effects on cholesterol metabolism. In contrast to rapamycin, which failed to elevate cholesterol levels in females under any nutritional regimen, trametinib effectively mitigated cholesterol loss in wild‐type females subjected to cholesterol‐deficient diets. Therefore, the amelioration of intestinal barrier integrity observed in wild‐type females treated with trametinib may still partially depend on the reduced cholesterol consumption resulting from the inhibition of reproduction.

## 3 Discussion

### 3.1 Intestinal Barrier Damage Caused by Reproductive‐Triggered Sterol Competition Limits Female Lifespan in Nutritionally Unbalanced Diet

The interplay between reproduction and survival manifests across numerous species, with observations that the ablation of germ cells or the inhibition of reproductive capabilities extends lifespan and enhances resistance to diverse environmental stressors (Flatt et al. 2008; Hsin and Kenyon 1999; Maklakov and Immler 2016; Min et al. 2012). Nevertheless, the perceived trade‐off between lifespan extension and reproductive output is not inevitable. Increasing evidence from genetic, pharmacological, and nutritional studies suggests that lifespan can be increased without compromising fecundity (Castillo‐Quan et al. 2016; Grandison et al. 2009; Maklakov and Immler 2016; Piper et al. 2017), thereby challenging the traditional “disposable soma” theory of aging, which posits a direct competition for resources between survival and reproduction (Kirkwood 1977; Shanley and Kirkwood 2000). In particular, recent studies have shown that simultaneous optimization of longevity and fecundity is feasible through precise adjustments in dietary macronutrients and micronutrients (Piper et al. 2017; Zanco et al. 2021), thus demonstrating that lifespan extension can be dissociated from diminished reproductive capacity, provided that the nutritional balance is meticulously maintained. Conversely, in the context of a nutritionally imbalanced diet, our studies showed that the competitive allocation of resources between reproductive processes and somatic maintenance emerge as critical limitations to lifespan, reaffirming the intricate relationship between dietary composition and biological aging mechanisms.

The effect of reproduction on longevity exhibits significant variation under differing nutritional conditions. Although eliminating reproduction induced nutritional competition consistently extended the female lifespan regardless of which component of the amino acids, sugars, vitamins, and cholesterol are omitted from food, the greatest lifespan reduction caused by reproduction occurs under conditions of cholesterol deficiency. This suggests that the decreased lifespan caused by reproduction in nutrient‐poor diets predominantly results from competition for limited cholesterol, and one explanation for this may be that females preferentially invest limited cholesterol into reproduction rather than somatic maintenance (Zanco et al. 2023).

The age‐related disruption of the intestinal barrier is a conserved pathological phenomenon observed across multiple species, including worms, fruit flies, rodents, and primates (Branca et al. 2019; Dambroise et al. 2016; Katz et al. 1987; Kavanagh et al. 2019; Rera et al. 2012; Salazar et al. 2023; Thevaranjan et al. 2017). Here, our study reveals that a decrease in lifespan attributable to dietary imbalances is not necessarily associated with compromised integrity of the intestinal barrier. Dietary amino acids or sucrose deficiency did not exacerbate the severity of intestinal barrier damage in wild‐type females, suggesting that the intake of these macronutrients, though vital for cellular metabolism and energetic processes, does not directly influence barrier integrity during aging. In contrast, the absence of either dietary vitamins or cholesterol significantly advanced the onset time of age‐related damage to the intestinal barrier, which supports the essential role of cholesterol in membrane structure and B vitamins in cellular repair processes (Krause and Regen 2014; Mikkelsen and Apostolopoulos 2019). Notably, however, in diets deficient in amino acids, sugars, or vitamins, *ovo* ^*D1*^ mutant groups accumulated a greater proportion of “smurf” flies over the entire life history than wild‐type females. It implies that while the suppression of reproductive activity delays mortality linked to the deprivation of these nutrients, it does not mitigate the progressive accumulation of intestinal barrier damage associated with extended lifespan. In contrast, in the context of cholesterol deficiency, the *ovo* ^*D*1^ mutation not only greatly delays the onset of intestinal barrier damage but also reduces its severity, thus suggesting that the alleviation of reproductive‐induced cholesterol competition enhances intestinal barrier function, which is pivotal for increasing female lifespan under conditions of malnutrition.

### 3.2 Rapamycin and Trametinib Improve Intestinal Barrier Function in a Cholesterol‐Deficient Diet

Previous studies have indicated that the life‐extending effects of rapamycin and trametinib are partially attributable to their effects on the protection of intestinal barrier integrity (Fan et al. 2015; Ureña et al. 2024). Here, our study shows that the effects of rapamycin and trametinib on lifespan and intestinal barrier function are directly related to the content of dietary cholesterol. Both drugs significantly extend the lifespan and improve the intestinal barrier integrity in females on a cholesterol‐deficient diet, but have relatively little effect on longevity and age‐related gut barrier damage on a cholesterol‐rich diet. In other words, what rapamycin and trametinib actually ameliorate is the intestinal barrier damage caused by cholesterol deficiency. This differential response might also account for the negligible impact of these drugs on lifespan and intestinal barrier function in males, who typically require much lower cholesterol levels and do not exhibit comparable age‐related intestinal barrier degradation as observed in females (Bjedov et al. 2010; Regan et al. 2016; Ureña et al. 2024; Wu, Yu, Cheng, et al. 2020).

Interestingly, although both rapamycin and trametinib reduce female fecundity, their role in improving intestinal barrier integrity appears to be independent, or at least not entirely reliant, on their reproductive inhibitory effects. This is evident as both pharmacological agents improve intestinal barrier function in infertile females under cholesterol‐deficient dietary conditions. A previous study has demonstrated that during the larval developmental stage, mTOR signaling inhibition induced autophagy activation promotes the mobilization of intracellular cholesterol and the synthesis of steroid hormones using cholesterol as a substrate, thereby accelerating the consumption of intracellular cholesterol (Texada et al. 2019). Consistent with this, we show that rapamycin decreases total cholesterol levels in *ovo* ^*D*1^ female flies. However, in contrast to its effects in *ovo* ^*D*1^ females, rapamycin does not significantly impact cholesterol levels in wild‐type females. A possible explanation is that reproductive inhibition caused by rapamycin leads to reduced cholesterol consumption for reproduction, thereby offsetting its cholesterol‐lowering effect. Nevertheless, since rapamycin fails to elevate cholesterol levels in females on a cholesterol‐deficient diet, its role in enhancing intestinal barrier integrity under these conditions is not attributable to reduced cholesterol consumption due to reproductive inhibition. In contrast, trametinib effectively mitigates the loss of stored cholesterol in wild‐type females on a cholesterol‐deprived diet, implying that its positive effect on intestinal barrier function may partially stem from reduced cholesterol consumption owing to reproductive inhibition. Therefore, one of the differences in the mechanisms by which rapamycin and trametinib improve intestinal barrier integrity is that they act through reproductive‐independent and partially reproductive‐dependent pathways, respectively. Since we found that dietary cholesterol deprivation during adulthood did not alter insulin signaling levels in females, future research on the specific downstream mechanisms by which the inhibition of mTOR or ERK signaling pathways ameliorates female intestinal barrier damage induced by cholesterol‐deficient diets would be of great interest.

Besides modulating cholesterol levels, other mechanisms may also mediate the amelioration of female intestinal barrier impairments by trametinib and rapamycin. Indeed, a recent study indicates that trametinib ameliorates aging‐associated gut pathology in females at least in part through its inhibition of RNA polymerase III (Pol III) in intestinal stem cells (Ureña et al. 2024). Furthermore, rapamycin and its target, mTORC1, have also been shown to regulate Pol III activity (Filer et al. 2017; Marshall et al. 2012; Michels et al. 2010). Collectively, our findings highlight the interplay of cholesterol‐dependent and cholesterol‐independent mechanisms, mediated by these pharmacological interventions, in enhancing intestinal barrier function.

## 4 Methods

### 4.1 Fly Stocks and Husbandry

The wild‐type stock *Dahomey* was collected in 1970 in Dahomey (now Benin). BL1,309 (ovo[D1] v[24]/C(1)DX, y[1] w[1] f[1]) were obtained from the Bloomington Stock Center and backcrossed into wildtype *Dahomey* for eight generations. *ovo* ^*D*1^ males were crossed with *Dahomey* females to obtain sterile *ovo* ^*D*1^ infertile females. All stocks were maintained at 25°C on a 12‐h: 12‐h light: dark cycle at 60% humidity using 1SY food (7 g/L agar; 50 g/L sucrose; 100 g/L yeast [Yeast brand: ANGEL 81000001]). For all experiments, flies were reared at standard larval density in 1SY food and eclosed adults were collected over a 12‐h period (Piper and Partridge 2016). Flies were mated for 48 h on 1SY food in all experiments before sorting into single sexes. 48 h‐mated females (except for Figure S1h–k where virgin females and mated males were included) were collected and transferred to FLYaa holidic medium (Piper et al. 2017) for lifespan and all physiology experiments. The sum mass of amino acids of the FLYaa medium was 10.74 g/L, equivalent to 9.7 g protein, and the carbohydrate content was 17.12 g/L. Therefore, the protein‐to‐carbohydrate ratio in the medium is approximately 1:1.8. All experimental flies were maintained at 25°C on a 12‐h:12‐h light:dark cycle at 60% humidity.

### 4.2 Lifespan and “Smurf” Analysis

Flies of single sex were randomly allocated to the experimental food treatments and housed in plastic fly vials containing food at a density of 10 females per vial (except for Figure S1h–k, where males were also tested at a density of 10 flies per vial), with 10 vials per condition (*n* = 100). All experimental food contained 0.2% Erioglaucine Disodium Salt (Sigma 861,146) except the medium for Figure 1b and Figure S3a. Flies were transferred to a fresh food source every 2–3 days, during which any deaths, censors and “smurf” flies were recorded. A fly was counted as a “smurf” flies when dye coloration could be observed outside of the digestive tract (Rera et al. 2012). Differences of lifespan and age‐related cumulation of “smurf” were assessed using the Cox regression model. Total proportion of “smurf” flies were assessed using Fisher's exact test.

### 4.3 Fecundity

The number of eggs laid in 24‐h periods (days 7–8) was counted in all experiment except Figure 1b, Figure S1a,i,j. In Figure 1b and Figure S1a,i,j, egg numbers in 24‐h periods were counted every 2–3 days from day 1 to day 17 (Figure 1b and Figure S1a) or day 1 to day 25 (Figure S1i,j), respectively. For each condition and each time point, 10 vials were counted. Each vial contained 10 flies. The same cohort of flies was used for each fecundity experiment and its corresponding lifespan and “smurf” assays. Egg‐laying differences were assessed by one‐way or two‐way ANOVA followed by Tukey's multiple comparisons test. For egg‐laying counts involving multiple days, a Generalized Linear Mixed Model (GLMM) was employed for the analysis.

### 4.4 Fecal Area and Food Intake Measurement

Fecal area and food intake was measured simultaneously using a modified apparatus based on the excreta quantification (EX‐Q) feeding assay (Wu, Yu, Cheng, et al. 2020). Briefly, flies were transferred to EX‐Q chambers with dye labeled FLYaa food (diameter = 6 mm) contained 0.2% Erioglaucine disodium salt (Sigma Aldrich, 861,146) and kept for 24 h at 25°C on a 12‐h:12‐h light:dark cycle at 60% humidity (5 flies/chamber, 10 chambers/treatment). After 24 h, flies were transferred out of the EX‐Q chambers and the excreta in the excreta collection plates were photographed, fecal area was analysis with Image J software. The dye in excreta were then dissolved in 2 mL 0.1% PBST. The absorbance of the liquid sample was then measured at 630 nm (Molecular Devices, FlexStation 3) and used for food intake calculation according to a standard curve prepared from stock solutions of pure dye.

### 4.5 Western Blots

Female flies were fed with experimental food for a duration of 10 days before the western blot experiments. Total protein of 10 female flies per replicate was extracted by homogenizing in 150 μL of RIPA buffer (Proteintech, PR20035). Protein extracts were then denatured 5 min at 100°C. Approximately 40 μg of protein extract was loaded per lane on polyacrylamide gel. Proteins were separated and transferred to polyvinylidene fluoride membrane. Membranes were blocked in 5% milk TBST for 1 h at room temperature, after which they were incubated with primary antibodies diluted in 5% BSA TBST overnight at 4°C. Primary antibodies used were as follows: α‐Tubulin (Proteintech, 66,031, 1:2000), Phospho‐Erk (Thr202/Tyr204) (Selleck, A5056, 1:1000), Total‐Erk (Selleck, A5029, 1:2000), Phospho‐Akt (Thr308) (Immunoway, YP0590, 1:2000), Total‐Akt (Immunoway, YM3618, 1:2000). Membranes were then washed with TBST for 5 min × 3 times, and incubated with secondary antibody (Goat Anti‐Mouse IgG HRP (BIOMIKY MK101A) or Goat Anti‐Rabbit IgG HRP (BIOMIKY MK103A), 1:10000) diluted in TBST for 1.5 h at room temperature, followed by washing with TBST for 5 min × 3 times. Blots were imaged using SCG‐W3000 Chemiluminescence imaging system (Servicebio) and analyzed using SWE Image Gray Analysis software.

### 4.6 Immunofluorescence

Female flies fed the experimental food for 10 days were starved for 4 h on pure agar medium, and then guts were dissected in ice‐cold PBS and immediately fixed in 4% formaldehyde for 20 min. Postfixation, guts were washed for 5 min × 3 times in PBST (0.1 M Phosphate Buffer/Triton‐X100) and blocked in 5% goat serum TBST for 30 min at room temperature, after which they were incubated with primary antibodies (4F3 anti‐discs large, DSHB, 1:50) diluted in 5% goat serum TBST overnight at 4°C. Then guts were washed with PBST for 5 min × 3 times and then re‐blocked in 5% goat serum TBST for 30 min at room temperature, after which they were incubated with secondary antibodies (Fluor 488 Goat Anti‐Mouse lgG, YEASEN, 33206ES60, 1:200) diluted in 5% goat serum TBST for 2 h at room temperature. After that, guts were washed with PBST for 10 min × 3 times and mounted in Antifade mounting media (Vector H‐1200). Images of anterior midgut were captured with a NIKON Eclipse Ti‐2 laser scanning confocal microscope.

### 4.7 Body Weight Measurement

Flies starved on pure agar medium for 6 h were anesthetized with CO_2_ and collected into 1.5 mL Eppendorf tubes for body weight measurement. For each treatment, 6 biological replicates were measured, each replicate contained 10 flies. Data were assessed by Multivariate ANOVA followed by Tukey's multiple comparisons (Figure 4k) or unpaired *t*‐test (Figure S2e) and reported as average body weight per female.

### 4.8 Cholesterol Assay

Total cholesterol levels of flies were determined using the Amplex Red Cholesterol Assay Kit (Invitrogen A12216). Flies were transferred to agar‐only medium for a 6‐h starvation to excrete all the food from their body. Post‐starvation, 10 flies per replicate were collected in a 1.5 mL Eppendorf tube (6 replicates per treatment) and homogenized in 200 μL of 1× buffer included in the kit, after which another 300 μL 1× buffer was added to the homogenized sample solution and vortex to dilute the sample solution. The diluted homogenate was centrifuged at 5000 rpm for 5 min and a 20 μL supernatant was assayed according to the instructions of the kit. Data were assessed by Multivariate ANOVA followed by Tukey's multiple comparisons (Figure 4j) or unpaired *t*‐test (Figure S2e).

### 4.9 Triacylglyceride Assay

Total triacylglyceride levels of flies were determined using the Triglyceride Assay Kit (Nanjing Jiancheng Bioengineering Institute, A110‐1‐1). Female flies fed in EX‐Q chambers (5 flies/chamber; 8 replicates) or standard fly vials (5 flies/vial; 8 replicates) for 10 days were transferred to agar‐only medium for a 6‐h starvation to excrete all the food in the body. Post‐starvation, 5 flies per replicate were collected in a 1.5 mL Eppendorf tube (8 replicates per treatment) and homogenized in 100 μL of EtOH. The homogenate was centrifuged at 4°C and 5000 rpm for 5 min, a 2.5 μL supernatant was assayed according to the instructions of the kit. Data were assessed by unpaired *t*‐test and reported as average triglyceride per female.

### 4.10 ATP Assay

ATP levels of flies were determined using the ATP Assay Kit (Solarbio, BC0305). 10 females flies per replicate were collected in a 1.5 mL Eppendorf tube (6 replicates per treatment). Add 100 μL of the extraction solution included in the kit to the sample and homogenize in an ice bath. The homogenate was centrifuge at 10,000 g for 10 min at 4°C, and the supernatant was transferred to another EP tube. A 50 μL of chloroform was added to the supernatant and vortex thoroughly. Centrifuge again at 10,000 g for 3 min at 4°C. A 20 μL supernatant was assayed according to the instructions of the kit. Data were assessed by unpaired *t*‐test.

### 4.11 RNA Isolation, Library Preparation, RNA Sequencing, and Differentially Expressed Genes Analysis

Female flies were fed with experimental food for a duration of 10 days prior to RNA extraction. Total RNA of 10 flies per replicate was extracted using the TRIzol reagent (Sangon Biotech, B511311). Each treatment consisted of 3 biological replicates. The purity and concentration of the RNA were assessed using the NanoDrop 2000 spectrophotometer (Thermo Scientific, USA), whereas its integrity was assessed using the Agilent 2100 Bioanalyzer (Agilent Technologies, Santa Clara, CA, USA). Following confirmation of RNA quality, libraries were constructed according to the manufacturer's instructions using the TruSeq Stranded mRNA LT Sample Prep Kit (Illumina, San Diego, CA, USA). These libraries were sequenced on an Illumina HiSeq X Ten platform by OE Biotech Co. Ltd. (Shanghai, China), producing 150 bp paired‐end reads. Raw reads of fastq format were processed using Trimmomatic (Bolger et al. 2014) and the low quality reads were removed to obtain the clean reads for subsequent analyses. The resultant clean reads were mapped to the *Drosophila* genome (Release_6_plus_ISO1_MT) using HISAT2 (Kim et al. 2015). Fragments per kilobase per million reads (FPKM) of each gene was calculated using Cufflinks (Trapnell et al. 2010), and read counts for each gene were obtained using HTSeqcount (Anders et al. 2015). Differential expression analysis was performed using the DESeq R package (2012), with genes displaying a P value < 0.05 and foldchange > 2 or foldchange < 0.5 classified as significantly differentially expressed. Hierarchical clustering of differentially expressed genes (DEGs) was performed to reveal expression patterns across different conditions and samples. Further, Gene Ontology (GO) enrichment analysis of DEGs were performed using R based on the hypergeometric distribution.

## Author Contributions

Q.W., G.Y., and M.Y. conceived the experiments; G.Y., K.C., and Q.W. performed experiments. All authors analyzed the data. G.Y., Q.W., and M.Y. wrote the manuscript. All authors provided final approval of the submitted version.

## Conflicts of Interest

The authors declare no conflicts of interest.

## Supporting information


Appendix S1.

## Data Availability

The data that support the findings of this study are available from the corresponding author upon reasonable request.
