# Supplementary material for: Reproductive‐Triggered Sterol Competition Exacerbates Age‐Related Intestinal Barrier Damage in Drosophila Females
Source: Aging Cell. 2025 Feb 7;24(6):e70011. doi: 10.1111/acel.70011 (PMC12151907; doi:10.1111/acel.70011)
Supplement: Supplementary file 1 — Appendix S1. [file ACEL-24-e70011-s001.zip › acel70011-sup-0001-AppendixS1/Supplemental Information.pdf]

Supplemental Information

Reproductive-triggered sterol competition exacerbates age-related intestinal barrier damage in *Drosophila* females

Guixiang Yu, Kejin Chen, Mingyao Yang, Qi Wu

Supplemental Data

Figure S1. Effects of dietary cholesterol deficiency on lifespan, fecundity, and intestinal barrier integrity of various strains of *Drosophila*.
Figure S2. Physical photos and use steps of the excreta quantification (EX-Q) box.
Figure S3. Effect of trametinib on female lifespan and intestinal barrier function in diets with various concentrations of cholesterol.
Figure S4. Effects of rapamycin and trametinib on intestinal pathological changes and diarrhea phenotype in females under cholesterol-deficient dietary conditions.
Table S1. Fecundity data for Figure 1b and Figure S1a.
Table S2. Lifespan data for Figure S1b.
Table S3. Smurf data for Figure 1g, g'.
Table S4. Lifespan data for Figure S1d.
Table S5. Smurf data for Figure S1e.
Table S6. Lifespan data for Figure S1f.
Table S7. Smurf data for Figure S1g.
Table S8. Smurf data for Figure 1h, h'.
Table S9. Lifespan data for Figure S1c.
Table S10. Lifespan data for Figure S1h.
Table S11. Fecundity data for Figure S1i-j.
Table S12. Smurf data for Figure S1k.
Table S13. Lifespan data for Figure 2a-g.
Table S14. Smurf data for Figure 2a-g.
Table S15. Fecundity data for Figure 2h.
Table S16. Smurf data for Figure 2i.
Table S17. Lifespan data for Figure 4a.
Table S18. Fecundity data for Figure 4b.
Table S19. Smurf data for Figure 4c-d.
Table S20. Lifespan data for Figure S3a.
Table S21. Fecundity data for Figure S3b.
Table S22. Lifespan data for Figure 4e.
Table S23. Fecundity data for Figure 4f.
Table S24. Smurf data for Figure 4g-h.
Table S25. Lifespan data for Figure S3d.
Table S26. Fecundity data for Figure S3e.
Table S27. Smurf data for Figure S3f-g.
Table S28. Cholesterol content data for Figure 4j.
Table S29. Body weight data for Figure 4k.
Table S30. Data of Cholesterol content / mg weight for Figure 4l.

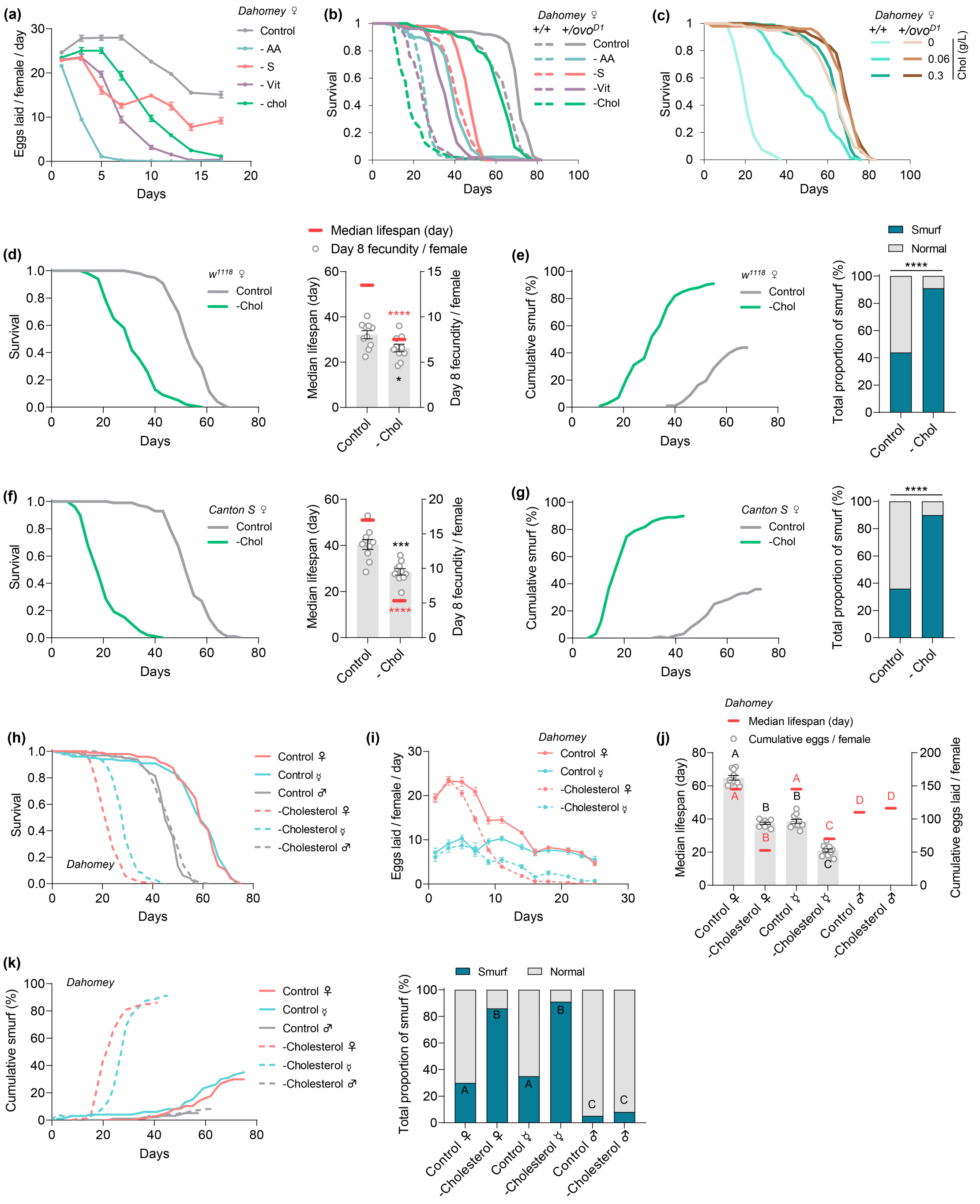

Figure S1. Effects of dietary cholesterol deficiency on lifespan, fecundity, and intestinal barrier integrity of various strains of *Drosophila*. (a) Removal of amino acids (AA), sucrose (S), B-group vitamins (Vit) or cholesterol (Chol) from the diet altered the fecundity in *Dahomey* wild-type females. (b) Lifespan curves of *Dahomey* wild-type (*+/+*) and *Dah; ovo*^{D1} (*+/ovo*^{D1}) females in diets omitting amino acids (AA), sucrose (S), B-group vitamins (Vit) or cholesterol (Chol). (c) Lifespan curves of *+/+* and *+/ovo*^{D1} females on diets with different cholesterol concentrations. (d-g) Dietary cholesterol deprivation decreased lifespan, fecundity and increased the ratio of age-related smurf in both *w*^{1118} (d, e) and *Canton S* (f, g) flies. (h-k) Effects of dietary cholesterol deprivation on lifespan (h), age-related fecundity (i, j), and cumulative percentage of smurf (k) in wild-type *Dahomey* flies. (n = 100 flies per treatment for lifespan and "smurf" assay, n = 10 biological replicates for egg laying in all trials, error bars

represent mean ± s.e.m. Lifespan differences were assessed using Cox regression model. Egg-laying differences were assessed by Generalized linear mixed model in panel a and i, by t test in panel d and f, by two-way ANOVA followed by Tukey's multiple comparisons in panel j. For panel e, g and k, age-related cumulation of 'smurf' were assessed using the Cox regression model, and the total proportion of "smurf" flies were assessed by Fisher's exact test. $^{***}p < 0.001$, $^{****}p < 0.0001$. For panel j and k, unique letters above or below the data indicate significant differences between conditions, $p < 0.05$. See statistical analysis of lifespan, fecundity and "smurf" data in Table S1, S2, S4-S7, S9-S12.

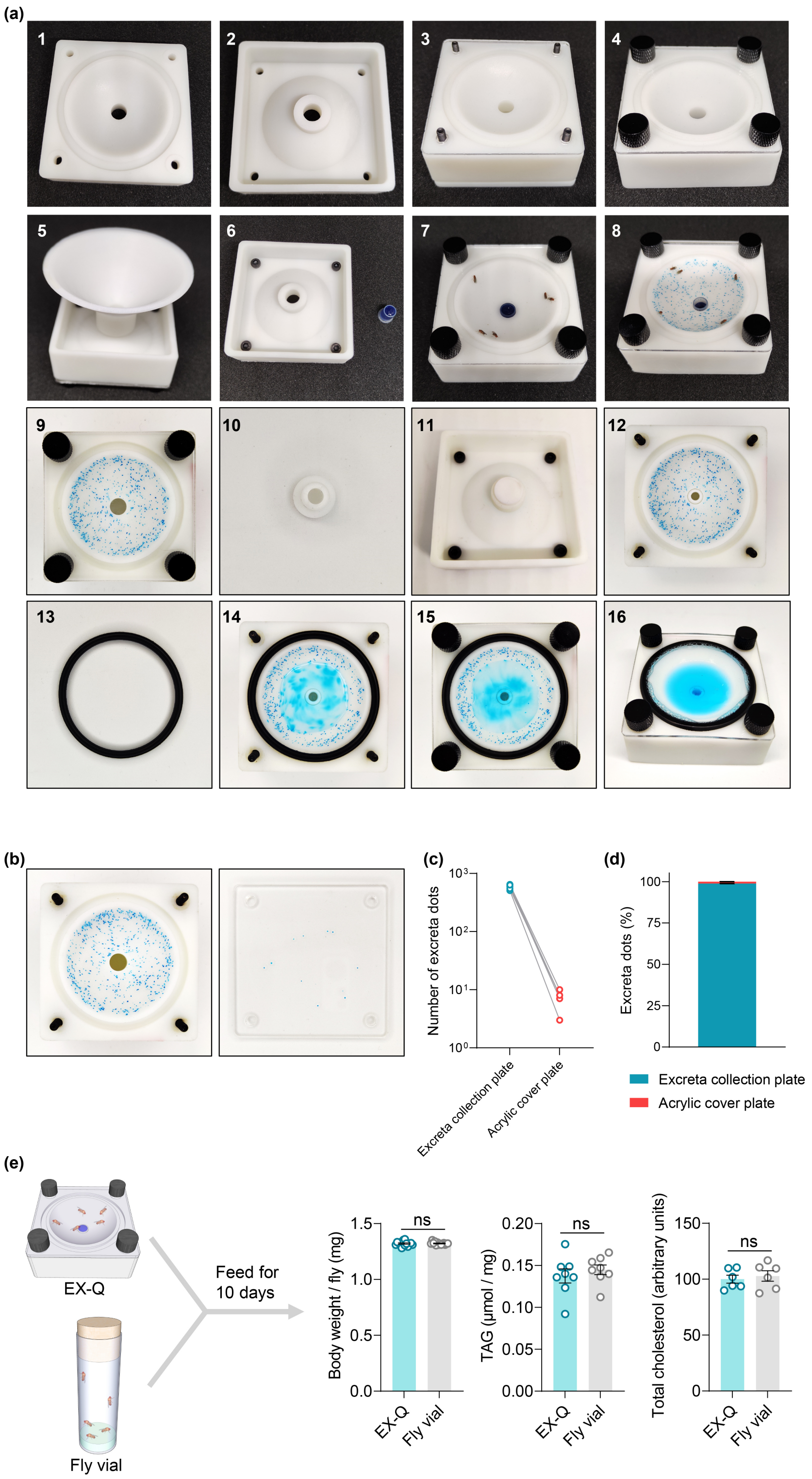

Figure S2. Physical photos and use steps of the excreta quantification (EX-Q) box. (a) Physical photos and use steps. (1 and 2) Front (1) and back (2) view of the 3D printed excreta collection plate. (3) Insert screws into the holes at the four corners of the excreta collection plate. The bottom of the excreta collection plate is supported by a 3D-printed base to prevent screws from falling out during assembly. Then install the acrylic cover plate over the excreta collection plate. (4) Install and tighten the nuts. (5) Turn the EX-Q box upside down and

transfer flies into EX-Q box using a 3D-printed funnel that fits into the excreta collection plate. (6) Remove the funnel from the EX-Q box and immediately stuff the food cup into the hole in the back of the excreta collection plate. (7) Turn the EX-Q box right side up, then place it in the incubator for 24 hours. (8 and 9) Remove the food cup and transfer flies out of the EX-Q box, photograph the excreta collection plate. (10 and 11) Insert a rubber plug (10) into the hole at the bottom of the excreta collection plate (11). (12) Remove the nuts and acrylic cover. (13 and 14) Place a rubber seal ring (13) in the annular groove, and then add 2mL PBST to the excreta collection plate (14). (15) Put the acrylic cover back on the plate and tighten the nuts. (16) Shake the EX-Q box to dissolve the dye in PBST. Food intake is calculated by measuring the absorbance of the solution at 630 nm. (b-d) Flies excrete the vast majority of their feces (> 98%) in the excreta collection plate, and only a very small amount of feces (< 2%) is excreted on the acrylic cover plate. (e) There were no significant differences in body weight, triglyceride and cholesterol contents between flies fed with EX-Q box and flies fed with standard *Drosophila* vials, assessed by t test.

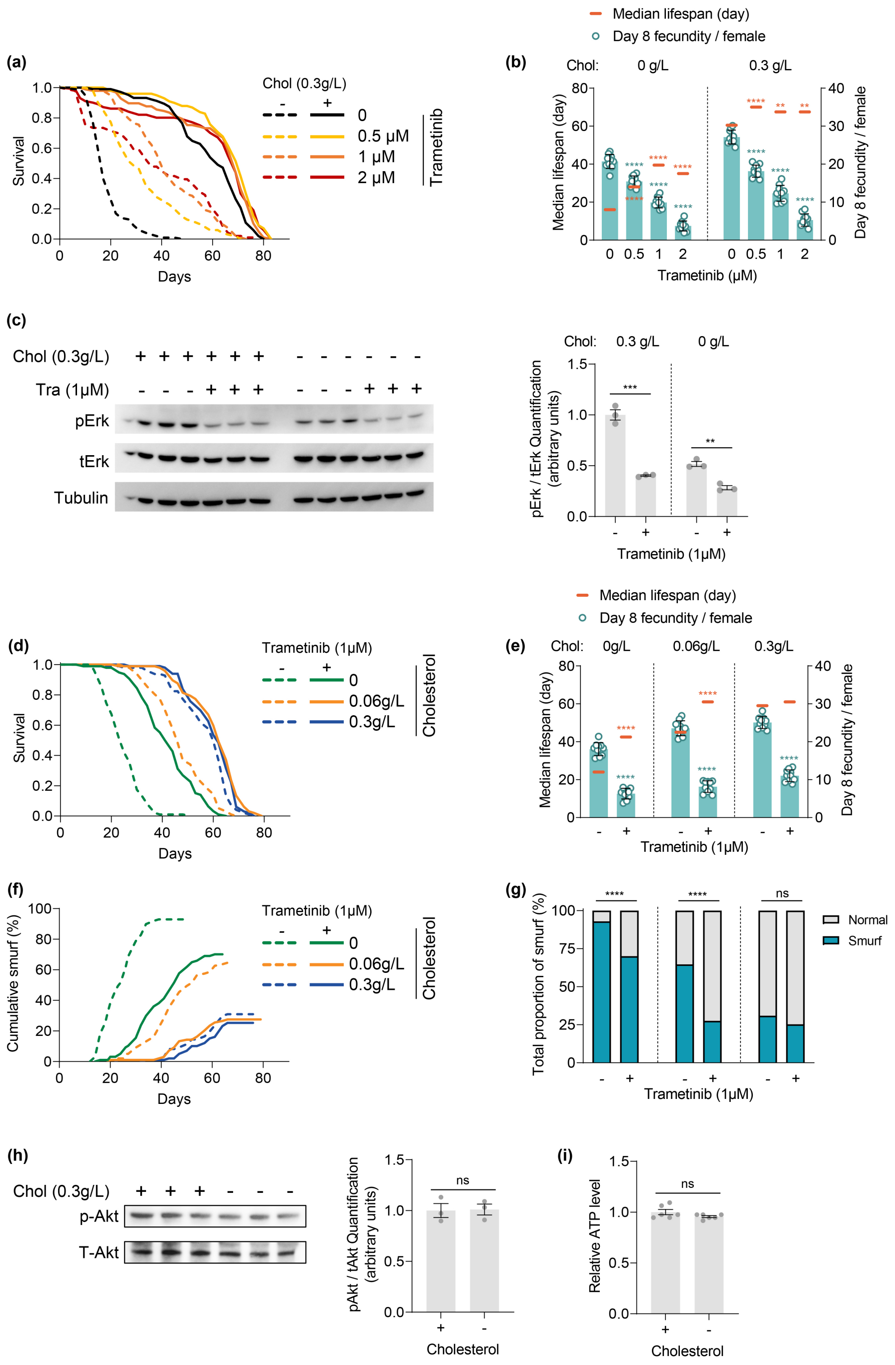

Figure S3. Effect of trametinib on female lifespan, fecundity and intestinal barrier function in diets with various concentrations of cholesterol. (a) Dose-dependent effect of trametinib on lifespan of wild-type females. (b) Dose-dependent effect of trametinib on fecundity and median lifespan of wild-type females. (c) 1μM of trametinib significantly reduced the phosphorylation level of Erk in wild-type females in both nutritionally complete and cholesterol dropout diets. (d) The life-extending effects of trametinib were maximized on a low-cholesterol diet (0 and 0.06 g/L). (e) Trametinib decreased the fecundity of wild-type females under dietary conditions at various concentrations of cholesterol. (f and g) Trametinib improved the intestinal barrier function of wild-type females under cholesterol-deficient dietary conditions (0 and 0.06 g/L), but had no effect on female intestinal barrier function under cholesterol-rich dietary condition (0.3 g/L). (h and i) Dietary cholesterol deprivation for

10 days had no significant effect on Akt phosphorylation level (h) and total ATP level (i) in wild-type females. (n = 100 flies per treatment for lifespan and “smurf” test, n = 10 biological replicates for egg laying in all trials, error bars represent mean ± s.e.m. Lifespan differences were assessed using Cox regression model. Egg-laying differences were assessed by two-way ANOVA followed by Tukey’s multiple comparisons. For panel c and h, western blotting data were assessed by unpaired t-test. For panel f and g, age-related cumulation of ‘smurf’ were assessed using the Cox regression model, and the total proportion of “smurf” flies were assessed by Fisher’s exact test. ** $p < 0.01$, *** $p < 0.001$, **** $p < 0.0001$. See statistical analysis of lifespan, fecundity and “smurf” data in Table S20, S21, S25-S27)

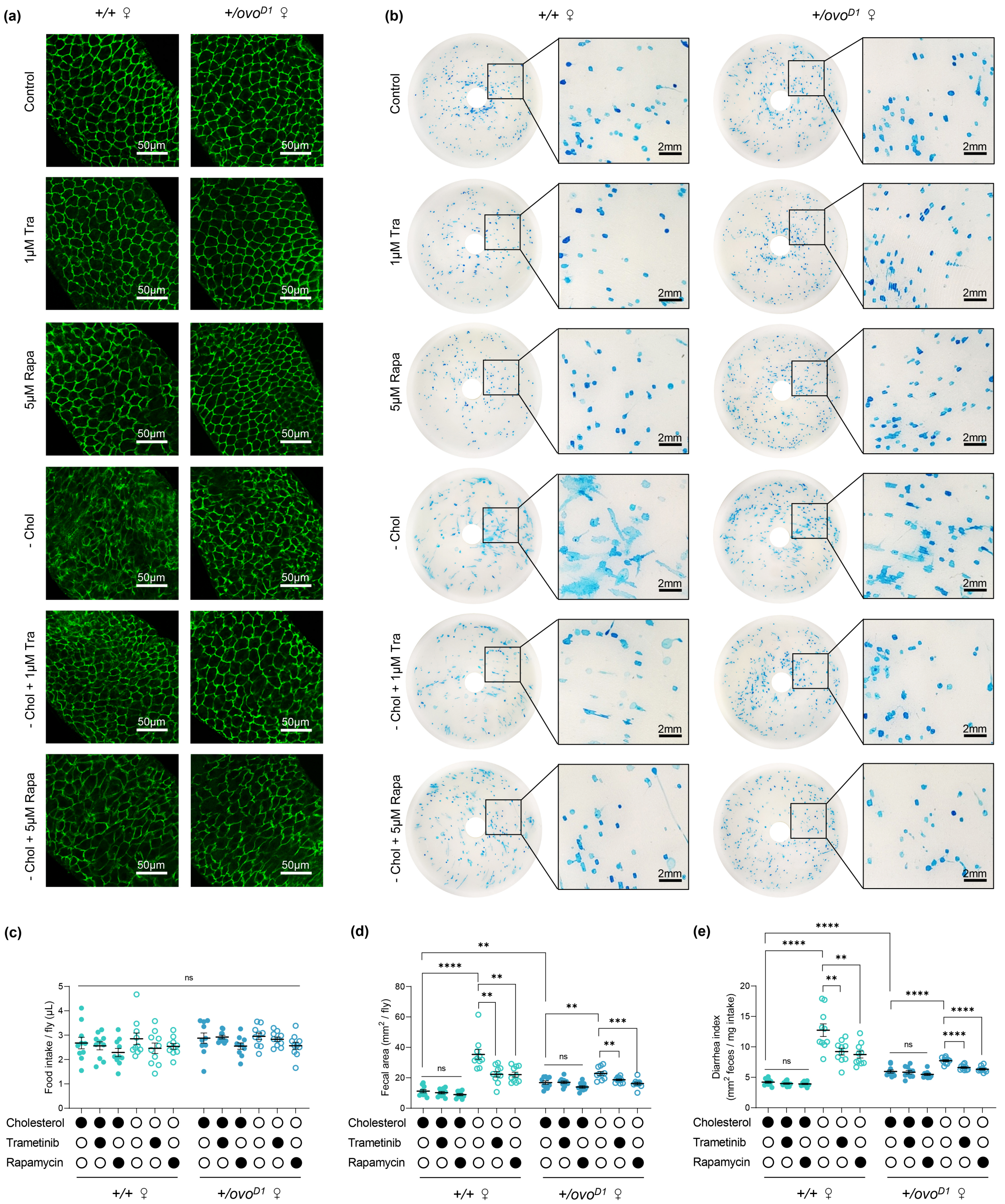

Figure S4. Effects of rapamycin and trametinib on intestinal pathological changes and diarrhea phenotype in females under cholesterol dropout dietary conditions. (a) Rapamycin and trametinib ameliorated intestinal pathological changes in 10-day-old wild-type (+/+) and ovo^{D1} (+/ovo^{D1}) females in cholesterol dropout diet. (b) Rapamycin and trametinib ameliorated the diarrhea phenotype in 10-day-old wild-type and ovo^{D1} females in cholesterol dropout diet. (c-e) Effect of rapamycin and trametinib on food intake (c), fecal area (d) and diarrhea index (e) of 10-day-old wild-type and ovo^{D1} females. (For panel c-e, solid black dots below the x-axis indicate the presence of cholesterol, rapamycin or trametinib in the medium, while open circles indicate their absence. For all excreta quantification and food intake measurement, n = 10 replicates in each treatment, each replicate contained 5 flies. Error bars represent mean ± s.e.m. For panel c-e, $**p < 0.01$, $****p < 0.0001$. Assessed

by unpaired t test.)

Table S1. Fecundity data for Figure 1b and Figure S1a.

	Genotype and Treatment	+/+ ♀ Control	+/+ ♀ -AA	+/+ ♀ -Sucrose	+/+ ♀ -Vit	+/+ ♀ -Chol
	Number of vials (N)	10	10	10	10	10
	Number of flies / vial	10	10	10	10	10
	Cumulative number of egg / fly	181.46	32.58	119.38	81.15	113.74
	SEM	1.5630	0.5470	1.9203	1.8577	3.0885
one-way ANOVA followed by Tukey's multiple comparisons (related to Figure 1b)	+/+ ♀ Control		<0.0001	<0.0001	<0.0001	<0.0001
Generalized linear mixed model (related to Figure S1a)	+/+ ♀ Control		<0.0001	<0.0001	<0.0001	<0.0001

Table S2. Lifespan data for Figure S1b.

	Genotype and Treatment	+/+ ♀ Control	+/+ ♀ -AA	+/+ ♀ -Sucrose	+/+ ♀ -Vit	+/+ ♀ -Chol	+/*ovo*^{D1} ♀ Control	+/*ovo*^{D1} ♀ -AA	+/*ovo*^{D1} ♀ -Sucrose	+/*ovo*^{D1} ♀ -Vit	+/*ovo*^{D1} ♀ -Chol
	Number of flies (N)	103	102	102	101	99	101	99	102	104	96
	median (d)	64.5	26.5	39.5	24	16.5	70.5	39.5	46.5	33.5	61.5
	mean (d)	61.6	26.6	41.7	25.3	19.1	69.4	39.3	46.7	36.0	60.1
log-rank test	+/+ ♀ Control		1.19E-41	6.79E-35	1.21E-43	2.81E-48	2.39E-11	8.93E-29	1.52E-31	3.33E-39	0.0648
	+/+ ♀ -AA			5.82E-30	0.5152	1.07E-10	2.20E-46	2.11E-23	1.38E-37	3.03E-22	2.67E-40
	+/+ ♀ -Sucrose				1.11E-29	4.27E-37	1.39E-42	0.1401	5.74E-06	3.67E-08	1.46E-32
	+/+ ♀ -Vit					4.56E-07	1.26E-47	1.59E-23	7.53E-41	2.76E-19	7.42E-42
	+/+ ♀ -Chol						9.25E-50	1.82E-29	6.53E-44	9.06E-29	2.41E-46
	+/*ovo*^{D1} ♀ Control							1.05E-36	4.63E-40	1.91E-44	5.63E-17
	+/*ovo*^{D1} ♀ -AA								1.29E-10	4.19E-05	1.34E-26
	+/*ovo*^{D1} ♀ -Sucrose									1.86E-24	1.20E-28
	+/*ovo*^{D1} ♀ -Vit										1.24E-37
	+/*ovo*^{D1} ♀ -Chol										
Cox regresssion	+/+ ♀ Control		1.97E-98	2.17E-35	3.41E-101	1.07E-134	1.20E-06	9.89E-36	2.73E-24	2.83E-55	0.1002
	+/+ ♀ -AA			6.83E-28	0.4798	0.0004	5.79E-124	7.09E-30	1.70E-39	5.44E-14	1.88E-87
	+/+ ♀ -Sucrose				1.85E-26	3.66E-49	3.15E-55	0.4500	0.0085	6.80E-05	2.25E-28
	+/+ ♀ -Vit					1.83E-05	2.27E-127	1.09E-28	2.84E-38	2.00E-12	1.06E-89
	+/+ ♀ -Chol						3.39E-162	3.96E-51	4.93E-65	4.92E-29	1.18E-121
	+/*ovo*^{D1} ♀ Control							1.24E-58	7.90E-42	6.79E-78	4.21E-10
	+/*ovo*^{D1} ♀ -AA								0.0743	3.95E-06	1.33E-27
	+/*ovo*^{D1} ♀ -Sucrose									7.46E-11	1.57E-18
	+/*ovo*^{D1} ♀ -Vit										1.50E-46
	+/*ovo*^{D1} ♀ -Chol										
	Genotype × AA	2.68E-06									
	Genotype × Sucrose	0.1047									
	Genotype × Vit	0.2169									
	Genotype × Chol	8.62E-21									

Note: +/+ ♀ represents wild-type Dahomey females.

Table S3. Smurf data for Figure 1g, g'.

	Genotype and Treatment	+/+ ♀ Control	+/+ ♀ -AA	+/+ ♀ -Sucrose	+/+ ♀ -Vit	+/+ ♀ -Chol	+/ovo^{D1} ♀ Control	+/ovo^{D1} ♀ -AA	+/ovo^{D1} ♀ -Sucrose	+/ovo^{D1} ♀ -Vit	+/ovo^{D1} ♀ -Chol
	Number of flies (N)	103	102	102	101	99	101	99	102	104	96
	Smurf	38	3	22	44	92	37	14	37	78	65
	Non-smurf	65	99	80	57	7	64	85	65	26	31
Cox regression analysis for age-related cumulative proportion of smurf (related to Figure 1g)	+/+ ♀ Control		0.0314	0.0424	1.06E-10	5.83E-53	0.6333	0.2559	1.16E-06	2.54E-24	1.12E-06
	+/+ ♀ -AA			0.0019	8.49E-07	6.50E-17	0.0491	0.0075	1.56E-05	2.98E-10	9.31E-05
	+/+ ♀ -Sucrose				8.06E-05	1.20E-34	0.0172	0.5369	0.0122	2.17E-13	0.1057
	+/+ ♀ -Vit					5.57E-25	9.83E-12	5.89E-05	0.1216	6.81E-05	0.0050
	+/+ ♀ -Chol						1.58E-54	1.80E-28	5.01E-31	1.99E-14	7.42E-40
	+/ovo^{D1} ♀ Control							0.1463	1.79E-07	9.94E-26	9.18E-08
	+/ovo^{D1} ♀ -AA								0.0045	5.35E-12	0.0400
	+/ovo^{D1} ♀ -Sucrose									2.69E-08	0.2615
	+/ovo^{D1} ♀ -Vit										2.13E-12
	+/ovo^{D1} ♀ -Chol										
	Genotype × AA	0.0075									
	Genotype × Sucrose	0.0122									
	Genotype × Vit	6.81E-05									
	Genotype × Chol	7.64E-40									
Fisher's exact test for total proportion of smurf (related to Figure 1g')	+/+ ♀ Control		<0.0001	0.0210	0.3918	<0.0001	0.9999	0.0002	0.9999	<0.0001	<0.0001
	+/+ ♀ -AA			<0.0001	<0.0001	<0.0001	<0.0001	0.0048	<0.0001	<0.0001	<0.0001
	+/+ ♀ -Sucrose				0.0010	<0.0001	0.0208	0.1996	0.0302	<0.0001	<0.0001
	+/+ ♀ -Vit					<0.0001	0.3891	<0.0001	0.3175	<0.0001	0.0009
	+/+ ♀ -Chol						<0.0001	<0.0001	<0.0001	<0.0001	0.0003
	+/ovo^{D1} ♀ Control							0.0003	0.9999	<0.0001	<0.0001
	+/ovo^{D1} ♀ -AA								0.0003	<0.0001	<0.0001
	+/ovo^{D1} ♀ -Sucrose									<0.0001	<0.0001
	+/ovo^{D1} ♀ -Vit										0.2754
	+/ovo^{D1} ♀ -Chol										

Table S4. Lifespan data for Figure S1d.

	Genotype and Treatment	w^{1118} ♀ Control	w^{1118} ♀ - cholesterol
	Number of flies (N)	98	100
	median (d)	53.5	29.5
	mean (d)	53.8	31.6
log-rank test		2.97E-36	
Cox regresssion		1.54E-28	

Table S5. Smurf data for Figure S1e.

	Genotype and Treatment	w^{1118} ♀ Control	w^{1118} ♀ - cholesterol
	Number of flies (N)	98	100
	Smurf	43	91
	Non-smurf	55	9
Cox regression analysis for age-related cumulative proportion of smurf (related to the left panel in Figure S1e)		8.58E-23	
Fisher's exact test for total proportion of smurf (related to the right panel in Figure S1e)		<0.0001	

Table S6. Lifespan data for Figure S1f.

	Genotype and Treatment	*Canton S* ♀ Control	*Canton S* ♀ - cholesterol
	Number of flies (N)	100	99
	median (d)	50.5	16
	mean (d)	53.5	20.3
log-rank test		1.78E-51	
Cox regresssion		1.70E-22	

Table S7. Smurf data for Figure S1g.

	Genotype and Treatment	*Canton S* ♀ Control	*Canton S* ♀ - cholesterol
	Number of flies (N)	100	99
	Smurf	36	89
	Non-smurf	64	10
Cox regression analysis for age-related cumulative proportion of smurf (related to the left panel in Figure S1g)		7.44E-13	
Fisher's exact test for total proportion of smurf (related to the right panel in Figure S1g)		<0.0001	

Table S8. Smurf data for Figure 1h, h'.

	Genotype and Treatment	+/+ ♀ 0g chol	+/+ ♀ 0.06g chol	+/+ ♀ 0.3g chol	+/ovo^{D1} ♀ 0g chol	+/ovo^{D1} ♀ 0.06g chol	+/ovo^{D1} ♀ 0.3g chol
	Number of flies (N)	99	99	99	99	101	101
	Smurf	91	72	38	71	29	28
	Non-smurf	8	27	61	28	72	73
Cox regression analysis for age-related cumulative proportion of smurf (related to Figure 1h)	+/+ ♀ 0g chol		9.27E-36	1.44E-54	4.90E-44	5.17E-59	5.30E-59
	+/+ ♀ 0.06g chol			5.39E-10	0.0071	1.22E-14	7.09E-15
	+/+ ♀ 0.3g chol				8.98E-05	0.0542	0.0397
	+/ovo^{D1} ♀ 0g chol					1.30E-08	7.37E-09
	+/ovo^{D1} ♀ 0.06g chol						0.8873
	+/ovo^{D1} ♀ 0.3g chol						
	Cholesterol concentration	1.89E-30					
	Genotype	8.88E-28					
	Cholesterol concentration × Genotype	3.74E-12					
Fisher's exact test for total proportion of smurf (related to Figure 1h')	+/+ ♀ 0g chol		0.0006	<0.0001	0.0004	<0.0001	<0.0001
	+/+ ♀ 0.06g chol			<0.0001	0.9999	<0.0001	<0.0001
	+/+ ♀ 0.3g chol				<0.0001	0.1778	0.1386
	+/ovo^{D1} ♀ 0g chol					<0.0001	<0.0001
	+/ovo^{D1} ♀ 0.06g chol						0.9999
	+/ovo^{D1} ♀ 0.3g chol						

Table S9. Lifespan data for Figure S1c.

	Genotype and Treatment	+/+ ♀ 0g chol	+/+ ♀ 0.06g chol	+/+ ♀ 0.3g chol	+/*ovo*^{D1} ♀ 0g chol	+/*ovo*^{D1} ♀ 0.06g chol	+/*ovo*^{D1} ♀ 0.3g chol
	Number of flies (N)	99	99	99	99	101	101
	median (d)	19.5	47.5	62.5	62.5	67	69.5
	mean (d)	21.4	49.8	62.7	61.9	67.0	67.8
log-rank test	+/+ ♀ 0g chol		2.70E-43	1.07E-50	2.82E-46	2.43E-48	2.23E-51
	+/+ ♀ 0.06g chol			1.14E-08	1.86E-08	3.05E-19	2.67E-20
	+/+ ♀ 0.3g chol				0.4299	1.23E-06	6.12E-08
	+/*ovo*^{D1} ♀ 0g chol					0.0008	0.0002
	+/*ovo*^{D1} ♀ 0.06g chol						0.5957
	+/*ovo*^{D1} ♀ 0.3g chol						
Cox regression	+/+ ♀ 0g chol		3.57E-44	3.34E-62	7.35E-66	6.17E-78	9.45E-80
	+/+ ♀ 0.06g chol			1.58E-07	6.28E-10	4.77E-19	1.55E-20
	+/+ ♀ 0.3g chol				0.2909	0.0001	2.13E-05
	+/*ovo*^{D1} ♀ 0g chol					0.0048	0.0012
	+/*ovo*^{D1} ♀ 0.06g chol						0.6811
	+/*ovo*^{D1} ♀ 0.3g chol						
	Cholesterol concentration	7.59E-14					
	Genotype	4.18E-33					
	Cholesterol concentration × Genotype	1.76E-32					

Note: +/+ ♀ represents wild-type Dahomey females.

Table S10. Lifespan data for Figure S1h.

	Genotype and Treatment	*Dahomey* ♀ 0.3g chol	*Dahomey* ♀ 0g chol	*Dahomey* ☿ 0.3g chol	*Dahomey* ☿ 0g chol	*Dahomey* ♂ 0.3g chol	*Dahomey* ♂ 0g chol
	Number of flies (N)	97	100	100	100	97	98
	median (d)	58.0	21.0	58.0	28.0	44.0	46.5
	mean (d)	58.6	22.7	56.9	28.6	45.0	46.0
log-rank test	*Dahomey* ♀ 0.3g chol		2.71E-48	0.8556	4.11E-46	2.75E-28	1.90E-25
	Dahomey ♀ 0g chol			7.70E-42	2.51E-13	3.40E-44	1.49E-45
	Dahomey ☿ 0.3g chol				6.59E-40	1.32E-25	1.24E-22
	Dahomey ☿ 0g chol					2.67E-38	7.47E-38
	Dahomey ♂ 0.3g chol						0.2108
	Dahomey ♂ 0g chol						
Cox regression	*Dahomey* ♀ 0.3g chol		4.31E-102	0.8653	8.52E-78	1.22E-26	7.81E-24
	Dahomey ♀ 0g chol			1.90E-102	1.15E-09	7.44E-55	8.02E-59
	Dahomey ☿ 0.3g chol				3.61E-78	4.97E-27	3.23E-24
	Dahomey ☿ 0g chol					2.35E-33	6.47E-37
	Dahomey ♂ 0.3g chol						0.2481
	Dahomey ♂ 0g chol						
	Gender	6.04E-23					
	Cholesterol	2.93E-73					
	Female mating status	0.8739					
	Gender × Cholesterol	8.84E-60					
	Female mating status × Cholesterol	3.37E-05					

Table S11. Fecundity data for Figure S1i-j.

	Genotype and Treatment	*Dahomey* ♀ 0.3g chol	*Dahomey* ♀ 0g chol	*Dahomey* ☿ 0.3g chol	*Dahomey* ☿ 0g chol
	Number of vials (N)	10	10	10	10
	Number of flies / vial	10	10	10	10
	Cumulative number of egg / fly	162.20	93.36	96.13	52.54
	SEM	3.5430	1.5620	3.2450	2.6030
two-way ANOVA followed by Tukey's multiple comparisons (related to Figure S1j)	Dahomey ♀ 0.3g chol		<0.0001	0.0001	<0.0001
	Dahomey ♀ 0g chol			0.9006	<0.0001
	Dahomey ☿ 0.3g chol				<0.0001
	Dahomey ☿ 0g chol				
	cholesterol	<0.0001			
	mating status	<0.0001			
	cholesterol × mating status	<0.0001			
Generalized linear mixed model (related to Figure S1i)	cholesterol	<0.0001			
	mating status	0.00011			
	cholesterol × mating status	<0.0001			

Table S12. Smurf data for Figure S1k.

	Genotype and Treatment	*Dahomey* ♀ 0.3g chol	*Dahomey* ♀ 0g chol	*Dahomey* ☿ 0.3g chol	*Dahomey* ☿ 0g chol	*Dahomey* ♂ 0.3g chol	*Dahomey* ♂ 0g chol
	Number of flies (N)	97	100	100	100	97	98
	Smurf	29	86	35	91	5	8
	Non-smurf	68	14	65	9	92	90
Cox regression analysis for age-related cumulative proportion of smurf (related to the left panel in Figure S1k)	*Dahomey* ♀ 0.3g chol		4.82E-34	0.4152	4.28E-28	0.0262	0.0269
Cox regression analysis for age-related cumulative proportion of smurf (related to the left panel in Figure S1k)	*Dahomey* ♀ 0g chol			1.30E-32	0.0069	1.92E-22	3.40E-28
Cox regression analysis for age-related cumulative proportion of smurf (related to the left panel in Figure S1k)	*Dahomey* ☿ 0.3g chol				1.28E-26	0.0076	0.0057
Cox regression analysis for age-related cumulative proportion of smurf (related to the left panel in Figure S1k)	*Dahomey* ☿ 0g chol					3.03E-19	4.82E-24
Cox regression analysis for age-related cumulative proportion of smurf (related to the left panel in Figure S1k)	*Dahomey* ♂ 0.3g chol						0.7260
Cox regression analysis for age-related cumulative proportion of smurf (related to the left panel in Figure S1k)	*Dahomey* ♂ 0g chol						
Cox regression analysis for age-related cumulative proportion of smurf (related to the left panel in Figure S1k)	Gender	0.0327					
Cox regression analysis for age-related cumulative proportion of smurf (related to the left panel in Figure S1k)	Cholesterol	2.69E-16					
Cox regression analysis for age-related cumulative proportion of smurf (related to the left panel in Figure S1k)	Female mating status	0.1151					
Cox regression analysis for age-related cumulative proportion of smurf (related to the left panel in Figure S1k)	Gender × Cholesterol	1.28E-06					
Cox regression analysis for age-related cumulative proportion of smurf (related to the left panel in Figure S1k)	Female mating status × Cholesterol	0.0347					
Fisher's exact test for total proportion of smurf (related to the right panel in Figure S1k)	*Dahomey* ♀ 0.3g chol		<0.0001	0.4520	<0.0001	<0.0001	0.0001
Fisher's exact test for total proportion of smurf (related to the right panel in Figure S1k)	*Dahomey* ♀ 0g chol			<0.0001	0.3757	<0.0001	<0.0001
Fisher's exact test for total proportion of smurf (related to the right panel in Figure S1k)	*Dahomey* ☿ 0.3g chol				<0.0001	<0.0001	<0.0001
Fisher's exact test for total proportion of smurf (related to the right panel in Figure S1k)	*Dahomey* ☿ 0g chol					<0.0001	<0.0001
Fisher's exact test for total proportion of smurf (related to the right panel in Figure S1k)	*Dahomey* ♂ 0.3g chol						0.5676
Fisher's exact test for total proportion of smurf (related to the right panel in Figure S1k)	*Dahomey* ♂ 0g chol						

Table S13. Lifespan data for Figure 2a-g.

					Cox regression				
	Genotype and Treatment	Number of flies (N)	median (d)	mean (d)	vs 0g cholesterol	vs 0.06g cholesterol	vs 0.3g cholesterol		
	0g cholesterol	100	18	20.1					
	0.06g cholesterol	96	36.5	39.3	1.29E-62				
	0.3g cholesterol	99	57.5	57.0	8.03E-125	1.39E-21			
								0.3g linoleic acid	1.5g linoleic acid
0g cholesterol +	0.06g linoleic acid	98	18	21.2	0.0740	3.25E-53	6.37E-113	0.6038	0.8253
	0.3g linoleic acid	97	18	20.6	0.2080	9.84E-56	4.68E-116		0.4602
	1.5g linoleic acid	100	22.5	19.7	0.0440	4.52E-51	1.92E-109		
								0.3g linolenic acid	1.5g linolenic acid
	0.06g linolenic acid	96	18	20.4	0.5597	2.22E-58	7.35E-119	0.4817	8.89E-05
	0.3g linolenic acid	99	18	20.8	0.1937	1.59E-55	1.04E-115		0.0011
	1.5g linolenic acid	97	22.5	24.7	5.54E-06	1.18E-37	1.53E-90		
								0.3g oleic acid	1.5g oleic acid
	0.06g oleic acid	99	18	20.3	0.3864	1.61E-58	5.08E-120	0.491	7.50E-14
	0.3g oleic acid	99	18	19.9	0.8600	5.72E-62	3.46E-124		9.26E-12
	1.5g oleic acid	96	11	14.2	0.0000	5.05E-109	8.29E-183		
								0.3g palmitoleic acid	1.5g palmitoleic acid
	0.06g palmitoleic acid	98	20	20.3	0.3209	8.36E-57	3.54E-117	0.8893	1.00E-55
	0.3g palmitoleic acid	100	18	20.5	0.3897	1.60E-58	6.10E-120		7.58E-56
	1.5g palmitoleic acid	99	2	5.9	1.91E-50	3.60E-188	1.36E-275		
								0.3g β-Sitosterol	1.5g β-Sitosterol
	0.06g β-Sitosterol	96	41	42.4	1.43E-70	0.1554	4.04E-16	2.45E-15	1.12E-19
	0.3g β-Sitosterol	100	57.5	58.1	1.21E-123	1.02E-20	0.7792		0.2113
	1.5g β-Sitosterol	101	57.5	57.0	2.46E-132	9.89E-26	0.3323		
								0.3g Stigmasterol	1.5g Stigmasterol
	0.06g Stigmasterol	97	36.5	38.1	5.05E-45	0.0003	1.19E-35	4.32E-46	5.06E-43
	0.3g Stigmasterol	99	60	59.7	2.92E-140	4.71E-31	0.0244		0.4698
	1.5g Stigmasterol	100	57.5	58.7	3.02E-136	3.78E-28	0.1217		
								0.3g Hydroxyecdysone	1.5g Hydroxyecdysone
	0.06g Hydroxyecdysone	97	18	20.2	0.1251	5.32E-57	3.81E-120	4.69E-05	1.79E-40
	0.3g Hydroxyecdysone	100	15.5	17.4	0.0108	6.52E-83	6.67E-153		3.80E-22
	1.5g Hydroxyecdysone	99	11	12.1	1.62E-33	1.12E-154	9.53E-236		

Table S14. Smurf data for Figure 2a-g.

	Genotype and Treatment	Number of flies (N)	Smurf	Non-smurf	Cox regression analysis for age-related cumulative proportion of smurf (related to Figure 2a-g)				
					vs 0g cholesterol	vs 0.06g cholesterol	vs 0.3g cholesterol		
	0g cholesterol	100	94	6					
	0.06g cholesterol	96	74	22	1.75E-33				
	0.3g cholesterol	99	26	73	1.17E-60	9.40E-17			
								0.3g linoleic acid	1.5g linoleic acid
0g cholesterol +	0.06g linoleic acid	98	93	5	0.2795	7.09E-29	3.10E-56	0.7122	2.23E-08
	0.3g linoleic acid	97	90	7	0.1494	5.18E-27	2.15E-54		1.80E-07
	1.5g linoleic acid	100	67	33	4.98E-11	7.62E-08	2.32E-31		
								0.3g linolenic acid	1.5g linolenic acid
	0.06g linolenic acid	96	90	6	0.4088	1.65E-29	7.48E-57	0.2917	0.0002
	0.3g linolenic acid	99	90	9	0.0589	3.13E-25	1.35E-52		0.0059
	1.5g linolenic acid	97	85	12	3.70E-06	5.92E-15	2.73E-41		
								0.3g oleic acid	1.5g oleic acid
	0.06g oleic acid	99	91	8	0.039	8.22E-25	3.19E-52	0.5994	0.0002
	0.3g oleic acid	99	89	10	0.127	1.51E-26	6.27E-54		0.0013
	1.5g oleic acid	96	77	19	0.0722	3.15E-39	3.18E-66		
								0.3g palmitoleic acid	1.5g palmitoleic acid
	0.06g palmitoleic acid	98	84	14	0.0098	7.82E-22	4.96E-49	0.1672	7.10E-06
	0.3g palmitoleic acid	100	92	8	0.2194	4.03E-28	1.69E-55		7.46E-08
	1.5g palmitoleic acid	99	20	79	9.10E-10	0.0538	7.15E-15		
								0.3g β-Sitosterol	1.5g β-Sitosterol
	0.06g β-Sitosterol	96	72	24	6.55E-37	0.442	1.01E-14	3.06E-16	2.37E-14
	0.3g β-Sitosterol	100	21	79	3.38E-59	3.29E-18	0.3781		0.2208
	1.5g β-Sitosterol	101	29	72	3.66E-62	1.92E-16	0.7326		
								0.3g Stigmasterol	1.5g Stigmasterol
	0.06g Stigmasterol	97	80	17	7.79E-25	0.0199	8.80E-23	9.89E-24	3.99E-23
	0.3g Stigmasterol	99	22	77	1.58E-59	6.37E-18	0.4842		0.5294
	1.5g Stigmasterol	100	26	74	3.44E-61	4.57E-17	0.9412		
								0.3g Hydroxyecdysone	1.5g Hydroxyecdysone
	0.06g Hydroxyecdysone	97	87	10	0.0136	3.22E-23	7.48E-51	7.54E-05	0.0139
	0.3g Hydroxyecdysone	100	94	6	0.1359	4.21E-42	9.30E-69		0.331
	1.5g Hydroxyecdysone	99	57	42	0.7473	4.42E-28	2.73E-55		

Table S15. Fecundity data for Figure 2h.

						one-way ANOVA followed by Tukey's multiple comparisons				
	Genotype and Treatment	Number of vials (N)	Number of flies / vial	Cumulative number of egg / fly	SEM	vs 0g cholesterol	vs 0.06g cholesterol	vs 0.3g cholesterol		
	0g cholesterol	10	10	14.08	0.63					
	0.06g cholesterol	10	10	17.56	0.71	0.4425				
	0.3g cholesterol	10	10	22.67	1.15	<0.0001	0.0113			
									0.3g linoleic acid	1.5g linoleic acid
0g cholesterol	0.06g linoleic acid	10	10	15.87	0.81	0.9991	0.9996	<0.0001	0.9991	<0.0001
	0.3g linoleic acid	10	10	14.09	0.97	>0.9999	0.4477	<0.0001		<0.0001
	1.5g linoleic acid	10	10	7.52	1.00	<0.0001	<0.0001	<0.0001		
									0.3g linolenic acid	1.5g linolenic acid
	0.06g linolenic acid	10	10	14.69	1.31	>0.9999	0.806	<0.0001	0.0091	<0.0001
	0.3g linolenic acid	10	10	9.51	1.02	0.0499	<0.0001	<0.0001		0.2411
	1.5g linolenic acid	10	10	5.65	0.71	<0.0001	<0.0001	<0.0001		
									0.3g oleic acid	1.5g oleic acid
	0.06g oleic acid	10	10	15.67	1.12	0.9999	0.998	<0.0001	0.9875	<0.0001
	0.3g oleic acid	10	10	13.51	0.63	>0.9999	0.1662	<0.0001		<0.0001
	1.5g oleic acid	10	10	6.04	0.89	<0.0001	<0.0001	<0.0001		
									0.3g palmitoleic acid	1.5g palmitoleic acid
	0.06g palmitoleic acid	10	10	12.73	0.88	>0.9999	0.0257	<0.0001	0.9017	<0.0001
	0.3g palmitoleic acid	10	10	15.36	1.02	>0.9999	0.9853	<0.0001		<0.0001
	1.5g palmitoleic acid	10	10	0.68	0.38	<0.0001	<0.0001	<0.0001		
									0.3g β-Sitosterol	1.5g β-Sitosterol
	0.06g β-Sitosterol	10	10	17.94	0.87	0.2387	>0.9999	0.0335	0.1181	>0.9999
	0.3g β-Sitosterol	10	10	22.16	1.29	<0.0001	0.0471	>0.9999		0.3488
	1.5g β-Sitosterol	10	10	18.52	0.76	0.0705	>0.9999	0.1337		
									0.3g Stigmasterol	1.5g Stigmasterol
	0.06g Stigmasterol	10	10	16.71	0.62	0.9033	>0.9999	0.0007	0.872	0.9996
	0.3g Stigmasterol	10	10	19.42	0.77	0.0056	0.9983	0.5851		>0.9999
	1.5g Stigmasterol	10	10	18.40	0.44	0.093	>0.9999	0.1032		
									0.3g Hydroxyecdysone	1.5g Hydroxyecdysone
	0.06g Hydroxyecdysone	10	10	12.73	0.88	>0.9999	0.0257	<0.0001	0.0001	<0.0001
	0.3g Hydroxyecdysone	10	10	6.24	0.79	<0.0001	<0.0001	<0.0001		>0.9999
	1.5g Hydroxyecdysone	10	10	4.89	0.67	<0.0001	<0.0001	<0.0001		

Table S16. Smurf data for Figure 2i.

					Fisher's exact test for total proportion of smurf (related to Figure 2i)				
	Genotype and Treatment	Number of flies (N)	Smurf	Non-smurf	vs 0g cholesterol	vs 0.06g cholesterol	vs 0.3g cholesterol		
	0g cholesterol	100	94	6					
	0.06g cholesterol	96	74	22	0.0009				
	0.3g cholesterol	99	26	73	<0.0001	<0.0001			
								0.3g linoleic acid	1.5g linoleic acid
0g cholesterol +	0.06g linoleic acid	98	93	5	0.9999	0.0003	<0.0001	0.5676	<0.0001
	0.3g linoleic acid	97	90	7	0.7807	0.0024	<0.0001		<0.0001
	1.5g linoleic acid	100	67	33	<0.0001	0.1521	<0.0001		
								0.3g linolenic acid	1.5g linolenic acid
	0.06g linolenic acid	96	90	6	1	0.0009	<0.0001	0.5931	0.2152
	0.3g linolenic acid	99	90	9	0.4353	0.0105	<0.0001		0.4961
	1.5g linolenic acid	97	85	12	0.1425	0.0608	<0.0001		
								0.3g oleic acid	1.5g oleic acid
	0.06g oleic acid	99	91	8	0.5928	0.0051	<0.0001	0.8055	0.0222
	0.3g oleic acid	99	89	10	0.3106	0.0199	<0.0001		0.0705
	1.5g oleic acid	96	77	19	0.0048	0.725	<0.0001		
								0.3g palmitoleic acid	1.5g palmitoleic acid
	0.06g palmitoleic acid	98	84	14	0.0616	0.1415	<0.0001	0.1804	<0.0001
	0.3g palmitoleic acid	100	92	8	0.7828	0.005	<0.0001		<0.0001
	1.5g palmitoleic acid	99	20	79	<0.0001	<0.0001	0.4003		
								0.3g β-Sitosterol	1.5g β-Sitosterol
	0.06g β-Sitosterol	96	72	24	0.0003	0.8659	<0.0001	<0.0001	<0.0001
	0.3g β-Sitosterol	100	21	79	<0.0001	<0.0001	0.4079		0.2537
	1.5g β-Sitosterol	101	29	72	<0.0001	<0.0001	0.7525		
								0.3g Stigmasterol	1.5g Stigmasterol
	0.06g Stigmasterol	97	80	17	0.0143	0.3751	<0.0001	<0.0001	<0.0001
	0.3g Stigmasterol	99	22	77	<0.0001	<0.0001	0.6191		0.6197
	1.5g Stigmasterol	100	26	74	<0.0001	<0.0001	0.9999		
								0.3g Hydroxyecdysone	1.5g Hydroxyecdysone
	0.06g Hydroxyecdysone	97	87	10	0.3058	0.0208	<0.0001	0.3058	<0.0001
	0.3g Hydroxyecdysone	100	94	6	1	0.0009	<0.0001		<0.0001
	1.5g Hydroxyecdysone	99	57	42	<0.0001	0.004	<0.0001		

Table S17. Lifespan data for Figure 4a.

	Genotype and Treatment	+/+ ♀ 0.3g chol	+/+ ♀ 0.3g chol 5μM Rapa	+/+ ♀ 0g chol	+/+ ♀ 0g chol 5μM Rapa	+/*ovo*^{D1} ♀ 0.3g chol	+/*ovo*^{D1} ♀ 0.3g chol 5μM Rapa	+/*ovo*^{D1} ♀ 0g chol	+/*ovo*^{D1} ♀ 0g chol 5μM Rapa
	Number of flies (N)	98	97	100	100	100	100	100	100
	median (d)	58.5	61.5	19	30.5	69	69	56	67
	mean (d)	58.2	61.8	21.2	34.8	69.3	70.3	58.1	65.9
log-rank test	+/+ ♀ 0.3g chol		0.0071	1.15E-48	3.66E-34	4.67E-22	5.92E-27	0.0869	3.26E-12
	+/+ ♀ 0.3g chol 5μM Rapa			3.46E-50	9.07E-43	1.22E-15	2.11E-19	0.6567	1.97E-05
	+/+ ♀ 0g chol				6.28E-26	5.97E-52	6.56E-52	2.41E-51	5.95E-52
	+/+ ♀ 0g chol 5μM Rapa					1.23E-48	2.20E-51	8.42E-30	5.13E-50
	+/*ovo*^{D1} ♀ 0.3g chol						0.7415	3.82E-14	3.36E-08
	+/*ovo*^{D1} ♀ 0.3g chol 5μM Rapa							7.26E-16	1.01E-09
	+/*ovo*^{D1} ♀ 0g chol								0.0006
	+/*ovo*^{D1} ♀ 0g chol 5μM Rapa								
Cox regression	+/+ ♀ 0.3g chol		0.0155	6.49E-78	2.37E-42	1.30E-21	7.77E-23	0.0102	2.18E-08
	+/+ ♀ 0.3g chol 5μM Rapa			2.27E-89	5.39E-54	1.45E-13	1.60E-14	0.8679	1.24E-03
	+/+ ♀ 0g chol				3.57E-24	3.53E-128	5.50E-130	9.21E-90	9.17E-106
	+/+ ♀ 0g chol 5μM Rapa					8.71E-95	8.74E-97	4.98E-54	4.64E-71
	+/*ovo*^{D1} ♀ 0.3g chol						0.7574	1.41E-13	1.20E-05
	+/*ovo*^{D1} ♀ 0.3g chol 5μM Rapa							1.68E-14	2.97E-06
	+/*ovo*^{D1} ♀ 0g chol								0.0020
	+/*ovo*^{D1} ♀ 0g chol 5μM Rapa								
	Genotype	1.39E-35							
	Cholesterol	1.34E-91							
	Rapamycin	3.29E-06							
	Genotype × Cholesterol	7.93E-38							
	Genotype × Rapamycin	2.03E-07							
	Cholesterol × Rapamycin	9.12E-09							

Note: +/+ ♀ represents wild-type Dahomey females.

Table S18. Fecundity data for Figure 4b.

	Genotype and Treatment	+/+ ♀ 0.3g chol	+/+ ♀ 0.3g chol 5μM Rapa	+/+ ♀ 0g chol	+/+ ♀ 0g chol 5μM Rapa
	Number of vials (N)	10	10	10	10
	Number of flies / vial	10	10	10	10
	Cumulative number of egg / fly	20.65	10.69	15.84	4.36
	SEM	0.6417	0.4929	0.8766	0.4388
two-way ANOVA followed by Tukey's multiple comparisons	+/+ ♀ 0.3g chol		<0.0001	<0.0001	<0.0001
	+/+ ♀ 0.3g chol 5μM Rapa			<0.0001	<0.0001
	+/+ ♀ 0g chol				<0.0001
	+/+ ♀ 0g chol 5μM Rapa				
	Cholesterol	<0.0001			
	Rapamycin	<0.0001			
	Cholesterol × Rapamycin	0.239			

Table S19. Smurf data for Figure 4c-d.

	Genotype and Treatment	+/+ ♀ 0.3g chol	+/+ ♀ 0.3g chol 5μM Rapa	+/+ ♀ 0g chol	+/+ ♀ 0g chol 5μM Rapa	+/*ovo*^{D1} ♀ 0.3g chol	+/*ovo*^{D1} ♀ 0.3g chol 5μM Rapa	+/*ovo*^{D1} ♀ 0g chol	+/*ovo*^{D1} ♀ 0g chol 5μM Rapa
	Number of flies (N)	98	97	100	100	100	100	100	100
	Smurf	29	30	91	71	30	32	60	35
	Non-smurf	69	67	9	29	70	68	40	65
Cox regression analysis for age-related cumulative proportion of smurf (related to Figure 4c)	+/+ ♀ 0.3g chol		0.8880	5.70E-60	1.83E-23	0.6506	0.9772	5.31E-06	0.5787
	+/+ ♀ 0.3g chol 5μM Rapa			6.63E-60	3.19E-23	0.5497	0.8630	9.00E-06	0.6796
	+/+ ♀ 0g chol				2.92E-24	3.09E-63	2.95E-62	2.41E-49	2.46E-61
	+/+ ♀ 0g chol 5μM Rapa					7.73E-26	1.14E-24	3.38E-12	2.33E-23
	+/*ovo*^{D1} ♀ 0.3g chol						0.6622	3.36E-07	0.3022
	+/*ovo*^{D1} ♀ 0.3g chol 5μM Rapa							2.50E-06	0.5502
	+/*ovo*^{D1} ♀ 0g chol								2.84E-05
	+/*ovo*^{D1} ♀ 0g chol 5μM Rapa								
	Genotype	0.0046							
	Cholesterol	7.54E-50							
	Rapamycin	0.3039							
	Genotype × Cholesterol	4.43E-26							
	Genotype × Rapamycin	1.05E-10							
	Cholesterol × Rapamycin	1.05E-10							
Fisher's exact test for total proportion of smurf (related to Figure 4d)	+/+ ♀ 0.3g chol		0.8769	<0.0001	<0.0001	0.9999	0.7594	<0.0001	0.4497
	+/+ ♀ 0.3g chol 5μM Rapa			<0.0001	<0.0001	0.9999	0.8794	<0.0001	0.5491
	+/+ ♀ 0g chol				<0.0001	0.0005	<0.0001	<0.0001	<0.0001
	+/+ ♀ 0g chol 5μM Rapa					<0.0001	<0.0001	0.1366	<0.0001
	+/*ovo*^{D1} ♀ 0.3g chol						0.8786	<0.0001	0.5461
	+/*ovo*^{D1} ♀ 0.3g chol 5μM Rapa							0.0001	0.7646
	+/*ovo*^{D1} ♀ 0g chol								0.0006
	+/*ovo*^{D1} ♀ 0g chol 5μM Rapa								

Note: +/+ ♀ represents wild-type Dahomey females.

Table S20. Lifespan data for Figure S3a.

	Genotype and Treatment	*Dahomey* ♀ 0g chol 0μM Tra	*Dahomey* ♀ 0g chol 0.5μM Tra	*Dahomey* ♀ 0g chol 1μM Tra	*Dahomey* ♀ 0g chol 2μM Tra	*Dahomey* ♀ 0.3g chol 0μM Tra	*Dahomey* ♀ 0.3g chol 0.5μM Tra	*Dahomey* ♀ 0.3g chol 1μM Tra	*Dahomey* ♀ 0.3g chol 2μM Tra
	Number of flies (N)	100	101	98	98	103	100	97	101
	median (d)	16	28	39.5	35	60.5	70	67.5	67.5
	mean (d)	18.9	32.9	42.6	37.9	59.2	66.4	62.3	59.6
log-rank test	*Dahomey* ♀ 0g chol 0μM Tra		2.91E-17	4.36E-33	3.44E-15	2.46E-49	2.69E-50	4.41E-44	2.49E-33
	Dahomey ♀ 0g chol 0.5μM Tra			0.0001	0.0061	5.87E-25	3.17E-35	1.04E-27	2.92E-23
	Dahomey ♀ 0g chol 1μM Tra				0.7590	6.78E-14	1.22E-25	1.89E-19	4.87E-17
	Dahomey ♀ 0g chol 2μM Tra					2.90E-12	7.60E-24	3.39E-19	2.83E-17
	Dahomey ♀ 0.3g chol 0μM Tra						2.44E-05	0.0066	0.0026
	Dahomey ♀ 0.3g chol 0.5μM Tra							0.1125	0.3753
	Dahomey ♀ 0.3g chol 1μM Tra								0.5260
	Dahomey ♀ 0.3g chol 2μM Tra								
Cox regression	*Dahomey* ♀ 0g chol 0μM Tra		9.22E-17	1.75E-28	7.71E-27	7.65E-62	2.64E-80	2.03E-72	4.92E-75
	Dahomey ♀ 0g chol 0.5μM Tra			0.0007	0.0014	2.15E-24	2.18E-39	3.75E-33	2.81E-35
	Dahomey ♀ 0g chol 1μM Tra				0.8500	3.83E-12	8.57E-24	6.12E-19	1.30E-20
	Dahomey ♀ 0g chol 2μM Tra					6.11E-13	3.39E-25	4.36E-20	6.70E-22
	Dahomey ♀ 0.3g chol 0μM Tra						0.0003	0.0200	0.0048
	Dahomey ♀ 0.3g chol 0.5μM Tra							0.1943	0.4134
	Dahomey ♀ 0.3g chol 1μM Tra								0.6217
	Dahomey ♀ 0.3g chol 2μM Tra								
	cholesterol	5.01E-64							
	trametinib	2.48E-21							
	cholesterol × trametinib	2.3917E-09							

Table S21. Fecundity data for Figure S3b.

	Genotype and Treatment	*Dahomey* ♀ 0g chol 0μM Tra	*Dahomey* ♀ 0g chol 0.5μM Tra	*Dahomey* ♀ 0g chol 1μM Tra	*Dahomey* ♀ 0g chol 2μM Tra	*Dahomey* ♀ 0.3g chol 0μM Tra	*Dahomey* ♀ 0.3g chol 0.5μM Tra	*Dahomey* ♀ 0.3g chol 1μM Tra	*Dahomey* ♀ 0.3g chol 2μM Tra
	Number of vials (N)	10	10	10	10	10	10	10	10
	Number of flies / vial	10	10	10	10	10	10	10	10
	Cumulative number of egg / fly	20.71	15.46	9.91	3.74	27.13	18.15	12.32	5.28
	SEM	0.5859	0.4520	0.4463	0.4160	0.5735	0.5097	0.6680	0.5187
two-way ANOVA followed by Tukey's multiple comparisons	*Dahomey* ♀ 0g chol 0μM Tra		<0.0001	<0.0001	<0.0001	<0.0001	0.0213	<0.0001	<0.0001
	Dahomey ♀ 0g chol 0.5μM Tra			<0.0001	<0.0001	<0.0001	0.0125	0.0018	<0.0001
	Dahomey ♀ 0g chol 1μM Tra				<0.0001	<0.0001	<0.0001	0.0372	<0.0001
	Dahomey ♀ 0g chol 2μM Tra					<0.0001	<0.0001	<0.0001	0.4477
	Dahomey ♀ 0.3g chol 0μM Tra						<0.0001	<0.0001	<0.0001
	Dahomey ♀ 0.3g chol 0.5μM Tra							<0.0001	<0.0001
	Dahomey ♀ 0.3g chol 1μM Tra								<0.0001
	Dahomey ♀ 0.3g chol 2μM Tra								
	cholesterol	<0.0001							
	trametinib	<0.0001							
	cholesterol × trametinib	<0.0001							

Table S22. Lifespan data for Figure 4e.

	Genotype and Treatment	+/+ ♀ 0.3g chol	+/+ ♀ 0.3g chol 1μM Tra	+/+ ♀ 0g chol	+/+ ♀ 0g chol 1μM Tra	+/ovo^{D1} ♀ 0.3g chol	+/ovo^{D1} ♀ 0.3g chol 1μM Tra	+/ovo^{D1} ♀ 0g chol	+/ovo^{D1} ♀ 0g chol 1μM Tra
	Number of flies (N)	97	99	98	97	100	99	101	98
	median (d)	63.5	66	17	35.5	73	75	59	73
	mean (d)	61.7	63.5	20.0	37.4	71.8	72.1	60.0	69.1
log-rank test	+/+ ♀ 0.3g chol		0.0093	8.55E-51	3.12E-22	1.21E-14	4.83E-17	0.9590	3.28E-10
	+/+ ♀ 0.3g chol 1μM Tra			2.52E-50	2.48E-25	8.84E-06	9.93E-09	0.0198	7.18E-05
	+/+ ♀ 0g chol				4.44E-23	8.43E-53	6.08E-50	1.40E-51	1.13E-50
	+/+ ♀ 0g chol 1μM Tra					4.49E-41	1.25E-38	1.34E-19	4.80E-33
	+/ovo^{D1} ♀ 0.3g chol						0.0306	1.06E-11	0.6820
	+/ovo^{D1} ♀ 0.3g chol 1μM Tra							1.97E-14	0.1297
	+/ovo^{D1} ♀ 0g chol								4.44E-09
	+/ovo^{D1} ♀ 0g chol 1μM Tra								
Cox regression	+/+ ♀ 0.3g chol		0.0087	8.63E-71	2.85E-23	6.13E-10	3.52E-14	0.7225	2.56E-10
	+/+ ♀ 0.3g chol 1μM Tra			2.76E-84	1.32E-34	2.00E-04	1.80E-07	0.0217	9.74E-05
	+/+ ♀ 0g chol				7.74E-30	8.16E-105	1.64E-112	5.30E-73	8.54E-105
	+/+ ♀ 0g chol 1μM Tra					1.04E-53	4.25E-61	8.79E-25	1.40E-53
	+/ovo^{D1} ♀ 0.3g chol						0.1012	2.91E-09	0.7906
	+/ovo^{D1} ♀ 0.3g chol 1μM Tra							1.48E-13	0.1701
	+/ovo^{D1} ♀ 0g chol								9.96E-10
	+/ovo^{D1} ♀ 0g chol 1μM Tra								
	Genotype	1.57E-18							
	Cholesterol	1.19E-84							
	Trametinib	1.94E-06							
	Genotype × Cholesterol	9.08E-38							
	Genotype × Trametinib	0.0001							
	Cholesterol × Trametinib	4.71E-13							

Note: +/+ ♀ represents wild-type Dahomey females.

Table S23. Fecundity data for Figure 4f.

	Genotype and Treatment	+/+ ♀ 0.3g chol	+/+ ♀ 0.3g chol 1μM Tra	+/+ ♀ 0g chol	+/+ ♀ 0g chol 1μM Tra
	Number of vials (N)	10	10	10	10
	Number of flies / vial	10	10	10	10
	Cumulative number of egg / fly	24.59	13.93	20.43	8.66
	SEM	0.5098	0.6370	0.6393	0.5822
two-way ANOVA followed by Tukey's multiple comparisons	+/+ ♀ 0.3g chol		<0.0001	0.0001	<0.0001
	+/+ ♀ 0.3g chol 1μM Tra			<0.0001	<0.0001
	+/+ ♀ 0g chol				<0.0001
	+/+ ♀ 0g chol 1μM Tra				
	Cholesterol	<0.0001			
	Trametinib	<0.0001			
	Cholesterol × Trametinib	0.3567			

Table S24. Smurf data for Figure 4g-h.

	Genotype and Treatment	+/+ ♀ 0.3g chol	+/+ ♀ 0.3g chol 1μM Tra	+/+ ♀ 0g chol	+/+ ♀ 0g chol 1μM Tra	+/*ovo*^{D1} ♀ 0.3g chol	+/*ovo*^{D1} ♀ 0.3g chol 1μM Tra	+/*ovo*^{D1} ♀ 0g chol	+/*ovo*^{D1} ♀ 0g chol 1μM Tra
	Number of flies (N)	97	99	98	97	100	99	101	98
	Smurf	34	27	88	64	31	28	69	38
	Non-smurf	63	72	10	33	69	71	32	60
Cox regression analysis for age-related cumulative proportion of smurf (related to Figure 4g)	+/+ ♀ 0.3g chol		0.1200	1.18E-55	6.68E-13	0.2143	0.1008	1.34E-05	0.8716
	+/+ ♀ 0.3g chol 1μM Tra			2.16E-58	1.67E-16	0.7163	0.9586	1.10E-08	0.1486
	+/+ ♀ 0g chol				1.06E-30	1.22E-60	1.46E-60	5.21E-45	1.24E-58
	+/+ ♀ 0g chol 1μM Tra					1.01E-16	1.97E-17	0.0003	3.80E-14
	+/*ovo*^{D1} ♀ 0.3g chol						0.6706	1.81E-08	0.2625
	+/*ovo*^{D1} ♀ 0.3g chol 1μM Tra							3.19E-09	0.1250
	+/*ovo*^{D1} ♀ 0g chol								2.72E-06
	+/*ovo*^{D1} ♀ 0g chol 1μM Tra								
	Genotype	0.2999							
	Cholesterol	1.82E-42							
	Trametinib	0.4851							
	Genotype × Cholesterol	2.55E-19							
	Genotype × Trametinib	9.82E-06							
	Cholesterol × Trametinib	3.19E-09							
Fisher's exact test for total proportion of smurf (related to Figure 4h)	+/+ ♀ 0.3g chol		0.2809	<0.0001	<0.0001	0.6495	0.3575	<0.0001	0.6568
	+/+ ♀ 0.3g chol 1μM Tra			<0.0001	<0.0001	0.6404	0.9999	<0.0001	0.0969
	+/+ ♀ 0g chol				<0.0001	<0.0001	<0.0001	0.0002	<0.0001
	+/+ ♀ 0g chol 1μM Tra					<0.0001	<0.0001	0.7634	0.0002
	+/*ovo*^{D1} ♀ 0.3g chol						0.7566	<0.0001	0.2969
	+/*ovo*^{D1} ♀ 0.3g chol 1μM Tra							<0.0001	0.1328
	+/*ovo*^{D1} ♀ 0g chol								<0.0001
	+/*ovo*^{D1} ♀ 0g chol 1μM Tra								

Note: +/+ ♀ represents wild-type Dahomey females.

Table S25. Lifespan data for Figure S3d.

	Genotype and Treatment	*Dahomey* ♀ 0g chol 0μM Tra	*Dahomey* ♀ 0g chol 1μM Tra	*Dahomey* ♀ 0.06g chol 0μM Tra	*Dahomey* ♀ 0.06g chol 1μM Tra	*Dahomey* ♀ 0.3g chol 0μM Tra	*Dahomey* ♀ 0.3g chol 1μM Tra
	Number of flies (N)	99	97	99	98	97	99
	median (d)	24	42.5	45	61	59	61
	mean (d)	25.1	42.2	47.4	60.9	57.2	60.3
log-rank test	*Dahomey* ♀ 0g chol 0μM Tra		3.93E-28	5.45E-39	3.55E-49	4.03E-46	3.78E-50
	Dahomey ♀ 0g chol 1μM Tra			0.0016	1.27E-26	5.42E-20	2.34E-26
	Dahomey ♀ 0.06g chol 0μM Tra				1.88E-17	7.11E-11	8.06E-17
	Dahomey ♀ 0.06g chol 1μM Tra					0.0020	0.3234
	Dahomey ♀ 0.3g chol 0μM Tra						0.0187
	Dahomey ♀ 0.3g chol 1μM Tra						
Cox regression	*Dahomey* ♀ 0g chol 0μM Tra		2.33E-33	2.32E-47	5.13E-89	3.58E-76	3.25E-86
	Dahomey ♀ 0g chol 1μM Tra			0.0007	1.57E-29	9.87E-20	1.02E-26
	Dahomey ♀ 0.06g chol 0μM Tra				6.68E-18	9.25E-10	2.63E-15
	Dahomey ♀ 0.06g chol 1μM Tra					0.0035	0.2994
	Dahomey ♀ 0.3g chol 0μM Tra						0.0507
	Dahomey ♀ 0.3g chol 1μM Tra						
	cholesterol	3.36E-55					
	trametinib	4.62E-32					
	cholesterol × trametinib	6.3158E-10					

Table S26. Fecundity data for Figure S3e.

	Genotype and Treatment	+/+ ♀ 0g chol 0μM Tra	+/+ ♀ 0g chol 1μM Tra	+/+ ♀ 0.06g chol 0μM Tra	+/+ ♀ 0.06g chol 1μM Tra	+/+ ♀ 0.3g chol 0μM Tra	+/+ ♀ 0.3g chol 1μM Tra
	Number of vials (N)	10	10	10	10	10	10
	Number of flies / vial	10	10	10	10	10	10
	Cumulative number of egg / fly	18.05	6.32	23.54	8.16	25.08	11.04
	SEM	0.5491	0.4409	0.6201	0.4824	0.4989	0.4905
two-way ANOVA followed by Tukey's multiple comparisons	+/+ ♀ 0g chol 0μM Tra		<0.0001	<0.0001	<0.0001	<0.0001	<0.0001
	+/+ ♀ 0g chol 1μM Tra			<0.0001	0.1381	<0.0001	<0.0001
	+/+ ♀ 0.06g chol 0μM Tra				<0.0001	0.3041	<0.0001
	+/+ ♀ 0.06g chol 1μM Tra					<0.0001	0.0031
	+/+ ♀ 0.3g chol 0μM Tra						<0.0001
	+/+ ♀ 0.3g chol 1μM Tra						
	cholesterol	<0.0001					
	trametinib	<0.0001					
	cholesterol × trametinib	0.0032					

Note: +/+ ♀ represents wild-type Dahomey females.

Table S27. Smurf data for Figure S3f-g.

	Genotype and Treatment	*Dahomey* ♀ 0g chol 0μM Tra	*Dahomey* ♀ 0g chol 1μM Tra	*Dahomey* ♀ 0.06g chol 0μM Tra	*Dahomey* ♀ 0.06g chol 1μM Tra	*Dahomey* ♀ 0.3g chol 0μM Tra	*Dahomey* ♀ 0.3g chol 1μM Tra
	Number of flies (N)	99	97	99	98	97	99
	Smurf	92	68	64	27	30	25
	Non-smurf	7	29	35	71	67	74
Cox regression analysis for age-related cumulative proportion of smurf (related to Figure S3f)	*Dahomey* ♀ 0g chol 0μM Tra		4.55E-25	2.16E-31	4.14E-44	1.32E-44	1.98E-45
	Dahomey ♀ 0g chol 1μM Tra			0.0976	4.27E-11	2.55E-10	3.84E-12
	Dahomey ♀ 0.06g chol 0μM Tra				9.92E-08	5.98E-07	1.23E-08
	Dahomey ♀ 0.06g chol 1μM Tra					0.6154	0.7074
	Dahomey ♀ 0.3g chol 0μM Tra						0.3760
	Dahomey ♀ 0.3g chol 1μM Tra						
	cholesterol	7.79E-20					
	trametinib	1.43E-15					
	cholesterol × trametinib	1.18E-05					
Fisher's exact test for total proportion of smurf (related to Figure S3g)	*Dahomey* ♀ 0g chol 0μM Tra		<0.0001	<0.0001	<0.0001	<0.0001	<0.0001
	Dahomey ♀ 0g chol 1μM Tra			0.4487	<0.0001	<0.0001	<0.0001
	Dahomey ♀ 0.06g chol 0μM Tra				<0.0001	<0.0001	<0.0001
	Dahomey ♀ 0.06g chol 1μM Tra					0.6390	0.7484
	Dahomey ♀ 0.3g chol 0μM Tra						0.4279
	Dahomey ♀ 0.3g chol 1μM Tra						

Table S28. Cholesterol content data for Figure 4j.

	Genotype and treatment	+/+ ♀ 0.3g chol	+/+ ♀ 0.3g chol 5μM Rapa	+/+ ♀ 0.3g chol 1μM Tra	+/+ ♀ 0g chol	+/+ ♀ 0g chol 5μM Rapa	+/+ ♀ 0g chol 1μM Tra	+/ovo^{D1} ♀ 0.3g chol	+/ovo^{D1} ♀ 0.3g chol 5μM Rapa	+/ovo^{D1} ♀ 0.3g chol 1μM Tra	+/ovo^{D1} ♀ 0g chol	+/ovo^{D1} ♀ 0g chol 5μM Rapa	+/ovo^{D1} ♀ 0g chol 1μM Tra
	Sample size (N)	6	6	6	6	6	6	6	6	6	6	6	6
	Mean cholesterol level (arbitrary units)	111.5	97.24	107.4	66.15	73.85	91.38	145.6	118.2	144	134	101.5	117.8
	SEM	1.501	4.208	2.252	2.385	1.725	2.816	4.833	2.57	1.46	2.417	2.267	1.7
Multivariate ANOVA followed by Tukey's multiple comparisons	+/+ ♀ 0.3g chol		0.0206	0.9953	7.91E-13	3.32E-12	0.0001	8.22E-11	0.8278	4.39E-10	1.13E-05	0.3069	0.8823
	+/+ ♀ 0.3g chol 5μM Rapa			0.2723	1.84E-09	4.79E-06	0.9247	7.90E-13	5.27E-05	7.90E-13	6.79E-12	0.9922	8.13E-05
	+/+ ♀ 0.3g chol 1μM Tra				8.57E-13	1.51E-10	0.0048	2.17E-12	0.1946	8.05E-12	1.89E-07	0.9251	0.2462
	+/+ ♀ 0g chol					0.6811	7.47E-07	7.90E-13	7.90E-13	7.90E-13	7.90E-13	2.39E-11	7.90E-13
	+/+ ♀ 0g chol 5μM Rapa						0.0013	7.90E-13	7.93E-13	7.90E-13	7.90E-13	6.05E-08	7.95E-13
	+/+ ♀ 0g chol 1μM Tra							7.90E-13	1.48E-07	7.90E-13	8.07E-13	0.2715	2.34E-07
	+/ovo^{D1} ♀ 0.3g chol								8.22E-08	0.9999	0.122	7.94E-13	5.19E-08
	+/ovo^{D1} ♀ 0.3g chol 5μM Rapa									4.46E-07	0.0059	0.0028	0.9999
	+/ovo^{D1} ♀ 0.3g chol 1μM Tra										0.2983	8.11E-13	2.82E-07
	+/ovo^{D1} ♀ 0g chol											4.67E-10	0.004
	+/ovo^{D1} ♀ 0g chol 5μM Rapa												0.0041
	+/ovo^{D1} ♀ 0g chol 1μM Tra												
	Genotype	1.22E-17											
	Cholesterol	1.87E-12											
	Rapamycin	1.63E-09											
	Trametinib	0.7258											
	Genotype × Cholesterol	0.0107											
	Genotype × Rapamycin	3.79E-07											
	Genotype × Trametinib	9.87E-05											
	Cholesterol × Rapamycin	0.0772											
	Cholesterol × Trametinib	0.122											

Note: +/+ ♀ represents wild-type Dahomey females.

Table S29. Body weight data for Figure 4k.

	Genotype and treatment	+/+ ♀ 0.3g chol	+/+ ♀ 0.3g chol 5μM Rapa	+/+ ♀ 0.3g chol 1μM Tra	+/+ ♀ 0g chol	+/+ ♀ 0g chol 5μM Rapa	+/+ ♀ 0g chol 1μM Tra	+/*ovo*^{D1} ♀ 0.3g chol	+/*ovo*^{D1} ♀ 0.3g chol 5μM Rapa	+/*ovo*^{D1} ♀ 0.3g chol 1μM Tra	+/*ovo*^{D1} ♀ 0g chol	+/*ovo*^{D1} ♀ 0g chol 5μM Rapa	+/*ovo*^{D1} ♀ 0g chol 1μM Tra
	Sample size (N)	6	6	6	6	6	6	6	6	6	6	6	6
	Body weight (mg / fly)	1.28	1.208	1.24	1.138	1.19	1.253	1.308	1.273	1.31	1.303	1.263	1.281
	SEM	0.0068	0.0091	0.0146	0.0166	0.0058	0.0117	0.0211	0.0117	0.0151	0.0163	0.0206	0.0202
Multivariate ANOVA followed by Tukey's multiple comparisons	+/+ ♀ 0.3g chol		0.0503	0.7619	5.157E-07	0.004	0.9794	0.9735	0.9999	0.9558	0.9935	0.9997	0.9999
	+/+ ♀ 0.3g chol 5μM Rapa			0.932	0.0644	0.9993	0.6087	0.0008	0.1108	0.0006	0.0018	0.2969	0.0417
	+/+ ♀ 0.3g chol 1μM Tra				0.0006	0.4484	0.9999	0.0825	0.912	0.0644	0.1377	0.9935	0.7189
	+/+ ♀ 0g chol					0.3881	6.591E-05	2.967E-09	1.745E-06	2.019E-09	6.928E-09	1.063E-05	3.929E-07
	+/+ ♀ 0g chol 5μM Rapa						0.1426	3.958E-05	0.0109	2.743E-05	8.801E-05	0.0425	0.0032
	+/+ ♀ 0g chol 1μM Tra							0.3076	0.998	0.2566	0.4381	0.9999	0.9694
	+/*ovo*^{D1} ♀ 0.3g chol								0.8901	0.9999	0.9999	0.6221	0.9824
	+/*ovo*^{D1} ♀ 0.3g chol 5μM Rapa									0.847	0.9558	0.9999	0.9999
	+/*ovo*^{D1} ♀ 0.3g chol 1μM Tra										0.9999	0.5547	0.9691
	+/*ovo*^{D1} ♀ 0g chol											0.7619	0.9963
	+/*ovo*^{D1} ♀ 0g chol 5μM Rapa												0.9993
	+/*ovo*^{D1} ♀ 0g chol 1μM Tra												
	Genotype	5.86E-06											
	Cholesterol	0.3666											
	Rapamycin	0.0525											
	Trametinib	0.2528											
	Genotype × Cholesterol	0.0811											
	Genotype × Rapamycin	0.2583											
	Genotype × Trametinib	0.0521											
	Cholesterol × Rapamycin	0.0159											
	Cholesterol × Trametinib	0.0080											

Note: +/+ ♀ represents wild-type Dahomey females.

Table S30. Data of Cholesterol content / mg weight for Figure 4I.

	Genotype and treatment	+/+ ♀ 0.3g chol	+/+ ♀ 0.3g chol 5μM Rapa	+/+ ♀ 0.3g chol 1μM Tra	+/+ ♀ 0g chol	+/+ ♀ 0g chol 5μM Rapa	+/+ ♀ 0g chol 1μM Tra	*+/ovo*^{D1} ♀ 0.3g chol	*+/ovo*^{D1} ♀ 0.3g chol 5μM Rapa	*+/ovo*^{D1} ♀ 0.3g chol 1μM Tra	*+/ovo*^{D1} ♀ 0g chol	*+/ovo*^{D1} ♀ 0g chol 5μM Rapa	*+/ovo*^{D1} ♀ 0g chol 1μM Tra
	Sample size (N)	6	6	6	6	6	6	6	6	6	6	6	6
	Relative cholesterol content / mg weight	87.07	80.46	86.64	58.22	62.08	73.04	111.4	92.92	109.9	102.9	80.42	92.05
	SEM	1.071	3.198	1.718	2.453	1.597	2.813	3.714	2.552	1.508	2.544	1.718	2.355
Multivariate ANOVA followed by Tukey's multiple comparisons	+/+ ♀ 0.3g chol		0.7176	0.9999	3.755E-10	3.291E-08	0.0054	7.191E-08	0.8459	3.814E-07	0.0009	0.7111	0.9418
	+/+ ♀ 0.3g chol 5μM Rapa			0.7948	8.002E-07	6.158E-05	0.5569	3.489E-11	0.0223	1.792E-10	6.174E-07	0.9999	0.0457
	+/+ ♀ 0.3g chol 1μM Tra				6.205E-10	5.456E-08	0.0081	4.339E-08	0.7773	2.307E-07	0.0006	0.7891	0.901
	+/+ ♀ 0g chol					0.9912	0.0025	7.897E-13	1.268E-12	7.897E-13	7.897E-13	8.323E-07	2.064E-12
	+/+ ♀ 0g chol 5μM Rapa						0.0744	7.897E-13	3.874E-11	7.897E-13	7.902E-13	6.392E-05	1.038E-10
	+/+ ♀ 0g chol 1μM Tra							7.978E-13	1.162E-05	8.298E-13	1.147E-10	0.5639	3.065E-05
	+/ovo^{D1} ♀ 0.3g chol								5.558E-05	0.9999	0.3538	3.358E-11	2.125E-05
	+/ovo^{D1} ♀ 0.3g chol 5μM Rapa									0.0003	0.146	0.0216	0.9999
	+/ovo^{D1} ♀ 0.3g chol 1μM Tra										0.636	1.723E-10	0.0001
	+/ovo^{D1} ♀ 0g chol											5.936E-07	0.0794
	+/ovo^{D1} ♀ 0g chol 5μM Rapa												0.0444
	+/ovo^{D1} ♀ 0g chol 1μM Tra												
	Genotype	1.81E-14											
	Cholesterol	5.69E-12											
	Rapamycin	1.36E-07											
	Trametinib	0.7794											
	Genotype × Cholesterol	0.0176											
	Genotype × Rapamycin	2.39E-06											
	Genotype × Trametinib	6.00E-04											
	Cholesterol × Rapamycin	0.3837											
	Cholesterol × Trametinib	0.4315											

Note: +/+ ♀ represents wild-type Dahomey females.
